# scEpiLock: A Weakly Supervised Learning Framework for *cis*-Regulatory Element Localization and Variant Impact Quantification for Single-Cell Epigenetic Data

**DOI:** 10.3390/biom12070874

**Published:** 2022-06-23

**Authors:** Yanwen Gong, Shushrruth Sai Srinivasan, Ruiyi Zhang, Kai Kessenbrock, Jing Zhang

**Affiliations:** 1 Center for Complex Biological Systems, University of California, Irvine, CA 92697, USA; yanweng@uci.edu; 2 Department of Biological Chemistry, School of Medicine, University of California, Irvine, CA 92697, USA; 3 Department of Computer Science, University of California, Irvine, CA 92697, USA; shushrruthsai@gmail.com (S.S.S.); ruiyiz5@uci.edu (R.Z.)

**Keywords:** scATAC-seq, *cis*-regulatory localization, deep learning, brain disorder

## Abstract

Recent advances in single-cell transposase-accessible chromatin using a sequencing assay (scATAC-seq) allow cellular heterogeneity dissection and regulatory landscape reconstruction with an unprecedented resolution. However, compared to bulk-sequencing, its ultra-high missingness remarkably reduces usable reads in each cell type, resulting in broader, fuzzier peak boundary definitions and limiting our ability to pinpoint functional regions and interpret variant impacts precisely. We propose a weakly supervised learning method, scEpiLock, to directly identify core functional regions from coarse peak labels and quantify variant impacts in a cell-type-specific manner. First, scEpiLock uses a multi-label classifier to predict chromatin accessibility via a deep convolutional neural network. Then, its weakly supervised object detection module further refines the peak boundary definition using gradient-weighted class activation mapping (Grad-CAM). Finally, scEpiLock provides cell-type-specific variant impacts within a given peak region. We applied scEpiLock to various scATAC-seq datasets and found that it achieves an area under receiver operating characteristic curve (AUC) of ~0.9 and an area under precision recall (AUPR) above 0.7. Besides, scEpiLock’s object detection condenses coarse peaks to only ⅓ of their original size while still reporting higher conservation scores. In addition, we applied scEpiLock on brain scATAC-seq data and reported several genome-wide association studies (GWAS) variants disrupting regulatory elements around known risk genes for Alzheimer’s disease, demonstrating its potential to provide cell-type-specific biological insights in disease studies.

## 1. Introduction

In eukaryotes, transcriptional regulation is essential to maintaining cell identity, responding to intra- and extra-cellular signals, and coordinating various gene activities [1], whereas its dysregulation can cause a broad range of diseases [2]. It is known that numerous *cis*-regulatory elements (CREs), such as enhancers and promoters, and their complex interactions orchestrate the precise spatiotemporal transcriptional regulation in a cell-type-specific manner. Variants in CRES can disturb the designed regulation systems, resulting in diseases. Therefore, accurate CRE localization and precise variant impact quantification are crucial to understanding transcriptional regulation and linking CREs’ genetic variations with phenotypic changes in human diseases. Fortunately, single-cell transposase-accessible chromatin using a sequencing assay (scATAC-seq) has recently emerged for the measurement of simultaneous chromatin accessibilities in thousands of individual cells [3,4], illuminating cellular heterogeneity at the epigenetic level and probing functional CREs at the single-cell resolution.

The general steps of analyzing an scATAC-seq include mapping reads to a reference genome, producing aligned reads, calling peaks, and returning a peak-by-cell matrix. The binary peak-by-cell matrix represents the peak accessibility for every cell. It can be used for downstream analyses including cell clustering, identifying differential accessible peaks, construction of *cis*-regulatory network, and inferring trajectories [5]. Most pipelines input a bam file to MACS2 [6] to call peaks, though the detailed steps to generate the peak-by-cell matrix can be different. SnapATAC generates a cell-by-bin count matrix first, then cells from the same cluster are pooled together, and MACS2 is used to call peaks for each cell type [7]. ArchR calls peaks with MACS2 from the coverage files, then reads in fragments from each chromosome and computes the overlaps with the peaks from the same chromosome [8]. Scasat [9] uses MACS2 on aggregated BAM files and peaks that are open in at least one single cell remaining in the peak-by-cell matrix. scAND [10] binarizes the peak-by-cell matrix and utilizes network diffusion methods to learn meaningful cell-type clusters. The peak-by-cell matrix can be aggregated into a peak-by-cluster matrix representing the binary peak accessibility for each cell type. Cell-type-specific analysis can be performed with this matrix to reveal the open chromatin characteristics for each cell type. For instance, we can identify motifs for each cell type by FIMO [11] or CentriMo [12]; predict *cis*-regulatory DNA interactions and co-accessibility by Cicero [13]; construct common or tissue-specific *cis*-regulatory interactions by JRIM [14]; etc.

Despite the continuous improvement in technology and analysis pipelines, two major challenges limit the application of scATAC-seq data to disease studies. First, unlike tissue-level sequencing where hundreds of millions of reads are mapped, the peak calling process in scATAC-seq experiments suffers from large amount of noise due to the sparsity of single-cell sequencing technologies, resulting in a fuzzy peak boundary and long peak length, which could lead to false-positive discoveries. For instance, most of the current scATAC-seq analysis pipelines yield low-resolution peaks with at least 501-bps [8], but the true functional parts of these CREs—e.g., binding sites of the transcription factors (TF)—are only 5–31 bp, with a mean of 9.9 bp for eukaryotes [15]. The large difference in region length reduces our statistical power in downstream analysis, e.g., CRE identification and disease-relevant variant mapping, and warrants the need to refine the call peaks in scATAC-seq. Second, after locating variants in functional CRE regions, a daunting question remains: how can we quantify variant impacts in a cell-type-specific manner to pinpoint key variants perturbating transcriptional regulatory mechanisms? Previous variant prioritizing methods fall into two main categories: (1) rank single nucleotide polymorphisms (SNPs) by the weighted integration of previous annotations for variants, e.g., FunSeq2 [16] and Combined Annotation-Dependent Depletion (CADD) [17]; (2) train a machine learning or deep learning classifier to identify functional SNPs, e.g., GWAVA [18] and CASAVA [19]. However, these methods were not designed for scATAC-seq and cannot evaluate variants in a cell-type-specific manner. There is an urgent need to develop a variant impact quantification method taking advantage of scATAC-seq data. 

Convolutional neural networks (CNNs) are a variant of deep neural networks using a weight-sharing strategy to capture local patterns that can usually reach high performance regarding classification tasks. There has been a growing interest to analyze sequencing data and dissect epigenomic features via CNN. For instance, DeepSEA [20], DanQ [21] and DeepATT [22] are deep learning methods with convolutional layers for predicting functions of non-coding sequences. Recent research in developing explainable deep learning models allows for the visualization of input regions with high-resolution details that are important for predictions. Gradient-weighted Class Activation Mapping (Grad-CAM) uses the gradients of any target concept flowing into the final convolutional layer to produce a localization map highlighting the important regions in the input for predicting the concept [23]. DECODE [24] uses Grad-CAM to condense the enhancer regions; AgentBind [25] incorporates Grad-CAM to predict the TF binding status. These studies inspire us to utilize CNN and Grad-CAM on scATAC-seq data for enhancing peaks and identifying functional variants.

**Figure 1:**
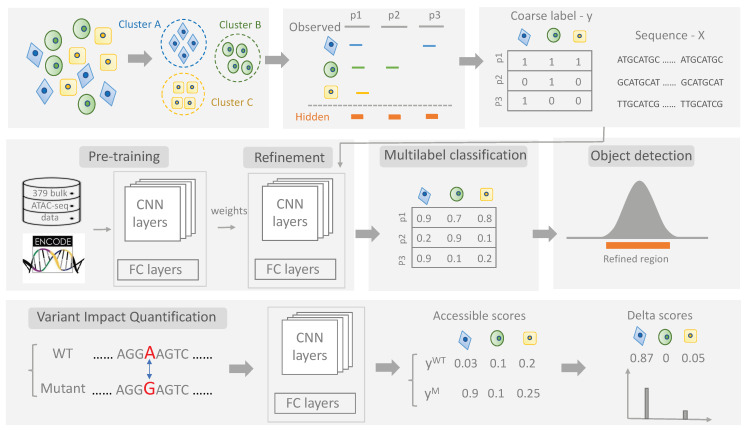
The overall design of the scEpiLock Method. The standard processing of scATAC-seq outputs coarse peaks and labels for each cell. scEpiLock takes the processed scATAC-seq cell x peak label matrix (y) and peak sequence (X) as inputs. First, the multi-label classifier module learns the biological pattern through CNN and makes multi-label predictions. Second, the object detection module refines the peak boundaries and localizes the potential *cis*-regulatory elements within the given peaks. Third, the variant impact quantification module computes the delta scores of candidate mutations and identifies their accessibility in a cell-type-specific manner.

We proposed a weakly supervised deep learning method, scEpiLock (Figure 1), to precisely locate CREs and quantify variant impacts in a cell-type-specific manner with three modules: (i) multi-label classifier module, (ii) object detection module, (iii) variant impact quantification module. First, the multi-label classifier module uses a convolutional neural network to make accessibility predictions on unseen sequences for each cell type. Then, we use Grad-CAM to produce a localization map to automatically highlight important regions in the predicted positive regions, which naturally refines the peaks’ boundaries and localizes the potential CREs. Lastly, the variant impact quantification module enables the computation of a base-wise accessibility score for wild type (WT), mutant, and their differences—the delta score. A high delta score indicates that the accessibility of given peak is sensitive to the variant.

To test the effectiveness of our scEpiLock, we applied the model on two public datasets: the 5k peripheral blood mononuclear cells (PBMC) from 10× genomics and the brain scATAC-seq [26]. We showed that our scEpiLock’s multi-label classifier module outperforms other models in predicting whether given peaks are accessible for certain cell types. In addition, the object detection module improves the peaks’ resolution by condensing the total peak length to only ⅓ of the original size. We also used scEpiLock to quantify the candidate variants’ impact on brain diseases. Its variant impact quantification module identified SNPs that largely alter peak accessibility and potentially disturb the disease-related gene expression.

## 2. Materials and Methods

In this study, we proposed a novel weakly supervised learning scheme, named scEpiLock, for the scATAC-seq data. It can: (1) predict accessible peaks for each cell type via a multi-label classifier module, (2) refine the *cis*-regulatory element boundary via an object detection module, and (3) identify important variants via the variant impact quantification module.

### 2.1. Data Processing

We applied scEpiLock on two publicly available datasets—5k PBMC data with 8 cell types and brain data with 6 cell types—to evaluate the model’s performance on scATAC-seq data. We also tested its transfer learning scheme using Encyclopedia of DNA Elements (ENCODE) bulk ATAC-seq data. 

#### 2.1.1. PBMC Data from 10× Genomics

We downloaded the raw 5k PBMC scATAC-seq readout from the 10× Genomics website, mapped the reads to the hg19 reference genome, and generated the cell x peak matrix via SnapATAC [7] following the pipeline tutorial. In the pre-processing step, we identified valid barcodes by requiring log10(UMI) to be between 3.5–5 and a promotor ratio within the range of 0.4 to 0.8. We removed unwanted chromosomes (e.g., chrM) and kept only chr1-22, chrX, and chrY. Next, we generated the cell-by-bin count matrix. The genome was segmented into uniform-sized bins (5k bp as default), and scATAC-seq profiles were represented by a cell-by-bin matrix. Each element indicated the number of sequencing fragments that had overlapped with a given bin in a certain cell. We further removed any cells with a bin coverage of less than 1000, as suggested by SnapATAC. In the end, we kept only the cell types with at least 200 cells to ensure the called peaks were reliable. As a result, 8 cell types had been identified, including 1486 CD14+ monocytes, 565 CD4 memory cells, 460 CD8 effectors, 349 CD4 naïve cells, 278 CD8 naïve cells, 278 pre-B cells, 261 double negative T cells, and 256 nature killer (NK) cells. A total of 114,538 peaks were identified, and each peak was accessible to at least one cell type. Among the called peaks, 53.1% of peaks (*n* = 60,834 peaks) were unique to one cell type, while 15.9% of peaks (*n* = 18,209 peaks) were shared among the 8 cell types. The rest of the peaks were shared between from two to seven cell types (Figure S1). 

#### 2.1.2. Brain scATAC-seq Data

We downloaded the brain cell x peak matrix [26]. The process to generate the file is detailed in the paper. Briefly, scATAC-seq was performed on 10 samples spanning the isocortex (*n* = 3), striatum (*n* = 3), hippocampus (*n* = 2), and substantia nigra (*n* = 2). The sequencing reads were mapped to the hg38 human reference genome. A total of 6 main brain cell types (excitatory neurons, inhibitory neurons, microglia, oligodendrocytes, astrocytes, and oligodendrocyte progenitor cells (OPCs)) were identified, ending with 221,062 peaks, 77.9% (*n* = 172,111 peaks) of which were specific to a single cell type (Figure S2).

#### 2.1.3. ENCODE Bulk ATAC-Seq Data

We used the ATAC-seq data on the ENCODE portal [27] (https://www.encodeproject.org/) (accessed on 1 March 2021) as the positive regions to train the transfer learning base model. By applying the filter of “ATAC-seq”, “Homo sapiens”, and “no perturbation”, we downloaded 379 bed files containing Irreproducibility Discovery Rate thresholded peaks (Table S1). We then merged all the peaks together, which had a total coverage of 0.56 billion bp. 

#### 2.1.4. Genome Features as Model Inputs

To prepare the inputs for scEpiLock, we kept a fixed length of the peaks at 1000 bp as suggested by previous studies [20,21,22] and then used one-hot encoding to format them into a 4 × 1000 binary matrix, with rows corresponding to A, G, C, and T, and the columns representing the peak length. Both forward and reverse complement strands were included in the training, which doubled the sample size. Each peak was paired with a multi-label vector, representing the accessible cell type label. For instance, if a given peak was accessible for two cell types, there would be two ones in the label vector at the corresponding cell type positions. The other cell types were labelled as zeros. The training, evaluation, and testing sets were randomly split by the ratio of 8:1:1.

#### 2.1.5. Negative Regions

To ensure our model recognized general non-accessible regions, we incorporated a human reference genome as the negative regions during training and testing. For the PBMC data, it was hg19, and for the brain data, it was hg38. This is to match the reference genomes used to map the corresponding scATAC-seq data. The human reference genome was downloaded and cut into 1000 bp genomic fragments. To train the base model for transfer learning, fragments overlapped with any ENCODE peaks were excluded, leaving 1127k fragments. The label of these negative fragments was a null matrix. For scATAC-seq data, negative fragments overlapping with any scATAC-seq peaks were also excluded. Then, 10,000 fragments were randomly sampled to be included. The labels of each negative control fragment were vectors of zeros, indicating their inaccessibility for any cell types.

### 2.2. scEpiLock Module 1—Multi-Label Classifier Module

This module is designed to make multi-label predictions of the peak accessibility for each cell type via CNN. Given a sequence of peaks, the output is a binary label vector, representing the chromatin status of all cell types. Specifically, 0 means that the peak is inaccessible for certain cell types, while 1 means it is accessible.

#### 2.2.1. Neural Network Architecture

**Figure 2:**
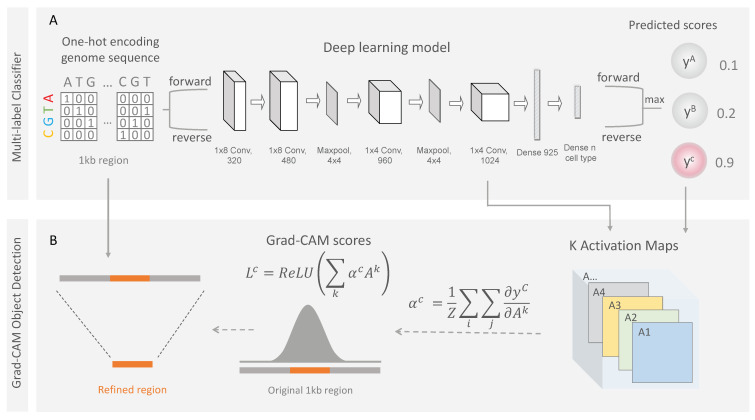
Details of the multi-label classifier module and object detection module. (**A**) An input sequence is first one-hot encoded into a 4-row bit matrix. Both the forward and reverse strands are fed into the CNN deep learning model. The architecture is composed of four convolutional layers, two polling layers, and two dense layers. The output is the sigmoid probability of each peak’s accessibility for each cell type. (**B**) For each positive prediction, we can use the Grad-CAM object detection module to refine the peak boundaries and localize the important non-coding regions. By superimposing activation maps (*A^k^*), weighted by an importance score (α*^c^*), the object detection module highlights the most salient sub-regions in making the final accessible decision.

As shown in Figure 2, scEpiLock uses a multilayer CNN model, which contains four convolutional layers, two pooling layers, and two dense, fully connected layers. The filters in the convolutional layers are trained to recognize the CREs. The dense, fully connected layers combine the learnt information and output the cell-type-specific peak accessible score. The model is organized into a sequential layer-by-layer structure, with each layer acting as a functional transformation. The detailed model architectures and hyperparameters used in this study can be found in Figure 2 and Note S1.

Our model contains four convolutional layers; each convolutional filter has the following form:

(1) $$\;A_{i}^{\left(l\right)}=\sum_{j}W_{i,j}^{\left(l\right)}\;\cdot \;A_{j}^{\left(l-1\right)}+B_{i}$$

In the equation, i and j represent the filter index; l is the layer index; W is the weight matrix; A is the activation map; B is the bias. 

The rectified linear activation function (ReLU) is added to the end of each convolutional layer to transform the output into non-negative values. This mitigates the issue of vanishing gradients and allows the models to converge faster.

(2) $$\;\;ReLU\left(x\right)=\{\begin{array}{c} x\;\;\;\;\;x\geq 0\\ 0\;\;\;\;\;\;x<0 \end{array}$$

The first several convolutional layers are designed to extract features from high-dimensional data for each cell type, e.g., the TF binding motifs. Then, max-pooling layers are used to reduce the number of parameters and abstract features trained in the previous convolutional layers. The results are pooled feature maps that highlight the most present feature in the patch. To avoid overfitting, we added dropouts to randomly set a proportion of the neuron activations to a value of 0. At the end, there are two dense, fully connected layers used to integrate the signals extracted from the previous convolutional layers. The first dense layer has 925 neurons, and the neuron number of the second dense layer is the same as the cell type number of each dataset, which outputs the accessible scores for each cell type. The dense layer is a nonlinear transformation function, which can be expressed as follows:

(3) Dense(X)=ReLU(WX+b)

A sigmoid function is used to obtain the different accessible probabilities of each given cell type. The prediction is scaled into the 0–1 range by the sigmoid function. Its formulation can be expressed as follows:

(4) $$\;Sigmoid\left(x\right)=1/\left(1+e^{-x}\right)$$

Multi-label classifier training configurations: the scEpiLock model was implemented using PyTorch [28] version 1.7.1, and a NVIDIA GeForce RTX 3090 graphic computing card (GPU) was used to train our models. All weights were initialized by randomly drawing from a uniform distribution as PyTorch’s default. We optimized the binary cross entropy loss by gradient descent via Adaptive Moment Estimation (Adam) at a learning rate of 5 × 10^−4^ and with a batch size of 64. The validation loss was evaluated at the end of each training epoch to monitor convergence. The data were split into 8:1:1 for training, validation, and testing. The model was fitted on the training set, and the hyper-parameters were evaluated on the validation set. After training, we selected the set of hyper-parameters that had the best performance on the validation set, which helped avoid overfitting. The final performance was reported on the unseen, independent test set. 

In some cases, the number of cells included in the scATAC-seq experiment might be small, resulting in a small number of peaks and high data sparsity. An alternative strategy for random initiation is transfer learning—first, train a model with ENCODE bulk ATAC-seq data and use the weights that gave the best performance as the initial weights to train the model. 

#### 2.2.2. Multi-Label Classifier Performance Evaluations

We used two metrics to evaluate the model’s performance on the test data set. The first was the Area Under Receiver Operating Characteristic curve (AUROC), which was created by plotting the true positive rate against the false positive rate at various threshold settings. Given the high negative proportion in the dataset, we also included the Area Under Precision Recall curve (AUPR), which plotted the area under the precision curve against the recall curve. AUPR was a more balanced metric than AURPC in accessing the model’s performance on an imbalanced dataset. For evaluating the performance on the test data set, the predicted probability for each sequence was computed as the max of the probability predictions for the forward and reverse complement sequencing pairs.

#### 2.2.3. Performance Benchmarking with Other Methods

For benchmark purposes, we also trained a DeepSEA model [20], a DanQ model [21], and a random forest (RF) model [29], for comparison with the scEpiLock. Both DeapSEA and DanQ are deep learning models developed for making functional predictions of genomic sequences. These two models are designed to predict 919 chromatin effect features. To make cell-type-specific multi-label predictions for each input peak, we changed the last fully connected layer from outputting n × 919 to n × m, where n is the number of peaks and m is the number of cell types. The implementation of DeepSEA and DanQ can be found in the GitHub repository we provided. Unlike the deep learning model, which took multi-dimension data, RF could only take a one-dimensional input per sequence. Thus, we converted the 4 × 1000 binary matrix to 4000 binary sequences by concatenating the values together. The max depth and minimum samples per leaf were set to 40 and 20, respectively, to avoid overfitting. The other parameters were kept as the default parameters from scikit-learn’s (v0.24.2) [30] Random Forest classifier function. 

### 2.3. scEpiLock Module 2—Object Detection for CRE Boundary Refinement

#### 2.3.1. Grad-CAM

We analogized the peak boundary localization problem as a classic object detection problem in the computer vision field and developed a weakly supervised learning scheme, Grad-CAM [23], to refine the very coarse peak definitions from the scATAC-seq data. Specifically, we used the gradients of any target (e.g., the positive peak label for a certain cell type) flowing into the last convolutional layer to produce a coarse localization map indicating the important regions in the peak for predicting the target (Figure 2). In turn, scEpiLock refined our peak annotations by reporting the most salient epigenomic regions, e.g., the TF binding sites, within the peaks as core regions. Specifically, the convolutional layers in scEpiLock were trained to identify the key regions within given peaks and retain spatial information which was lost in the fully connected layers. Thus, the last convolutional layer had the best balance between high-level representation and detailed spatial information. The gradient information flowing into the last convolutional layer of the CNN provided the necessary values for each neuron to predict cell-type-specific accessibility. Thus, we calculated the score’s gradient for the cell type c, $y^{c}$, with respect to each feature map activation $A^{k}$ of a convolutional layer, i.e., ($\frac{\partial y^{c}}{\partial A_{ij}^{k}}$). These gradients were global-average pooled to obtain the neuron importance weights ($\alpha_{k}^{c}$)

(5) $$\;\alpha_{k}^{c}=\frac{1}{Ζ}\underset{i}{\sum }\underset{j}{\sum }\frac{\partial y^{c}}{\partial A_{ij}^{k}}$$

To obtain the Grad-CAM score, we performed a weighted combination of the forwarded activation maps followed by an ReLU.

(6) $$\;\;L_{Grad-CAM}^{c}=ReLU\left(\overset{K}{\underset{k=1}{\sum }}\alpha_{k}^{c}A^{k}\right)$$

The output of the Grad-CAM maps was a 1D map with the size of the last convolutional feature maps (57). To assess the position-wise feature importance of the original 1 kb input fragment, we upscale the 1D map back to a length of 1000. Thus, one Grad-CAM value represented the region importance of 17.5 bp bin (1000/57 = 17.5). This perfectly covered one TF binding site. To refine our predictions, we used the Grad-CAM score to select a subset of the 17.5 bp bins with higher scores. Here, our cut-off was the 80th percentile of all the Grad-CAM scores computed. Such weakly supervised learning schemes increased our model’s interpretability by revealing and visualizing the process of decision making in our network.

#### 2.3.2. Conservation Scores for Refined Regions

We compared the cross-species conservation scores of the refined vs. raw peaks to test whether scEpiLock could detect true functional regions, as these scores were strong functionality indicators [31,32]. Specifically, we downloaded 100-way PhastCons [33] and calculated the averaged conservation scores per raw peak vs. scEpiLock’s refinements. A one-sided *t* test was used to calculate the *p*-values. 

### 2.4. scEpiLock Module 3—Variant Impact Quantification

To identify important variants that altered a cell type’s chromatin accessibility, we used a previously trained scEpiLock multi-label classifier to predict the peak accessible probability (accessible score) for the WT and candidate mutants. We then computed the absolute difference between the WT and candidate mutants to generate the delta score. A higher delta score indicated the variant has a larger functional impact for a particular cell type.

#### 2.4.1. GWAS Data Used and SNP Extraction

We downloaded a list of 930 putative disease-relevant SNPs for Alzheimer’s (AD) and Parkinson’s (PD). The criteria and process of selecting the SNPs are detailed in the original paper [26], and the full list of SNPs can be found in their Supplementary Table S2. We first trained our scEpiLock model on the brain scATAC-seq data. Then, we used the trained model to predict the cell-type accessibility probability (accessible score) of both the WT and mutant for each cell type. Functional SNPs were those with large accessible score differences between the WTs and mutants.

#### 2.4.2. Sequencing Tracks

All sequencing tracks were created using the UCSC Genome Browser [34] and shared the same x axis with the hg38 reference genome. The scATAC-seq, SNP, co-accessibility, and HiChIP tracks were custom tracks, while the other tracks were selected from the Genome Browser. The track of the scATAC-seq was the brain scATAC-seq cell-type-specific peaks. The SNP tracks of rs1237999, rs636317, and rs10769263 were added in the format of a Personal Genome SNP. The co-accessibility-based peak links were created by Cicero [13] using the brain scATAC-seq peaks. Both the co-accessibility and the HiChIP data were downloaded from the paper’s Supplementary Data 9 [26] and formatted as interactive tracks viewed in the Genome Browser.

## 3. Results

scEpiLock is a deep learning framework that refines scATAC-seq peaks and quantifies variant impacts in a cell-type-specific fashion, which contains three major modules: multi-label classifier, object detection, and variant impact quantification (Figure 1 and Figure 2). The scEpiLock model uniquely provides an accurate peak accessible probability, refines peak boundaries, and quantifies impactful variants. To demonstrate it, we first utilized the readout from scATAC-seq to train multi-label classifiers, which made accurate multi-label predictions for each peak (3.1). Then, we incorporated the object detection module to define high-resolution peak boundaries, reducing the total peak length to 1/3 of its original length while achieving higher conservation scores (3.2). Lastly, we evaluated GWAS SNPs in AD and PD by computing the accessible scores and identified functional SNPs that may directly affect TF binding (3.3).

### 3.1. The Multi-Label Classifier in scEpiLock Precisely Predicts Accessible Peaks

scATAC-seq can have very high throughputs, generating millions of reads at once. Despite this, their high data sparsity remains due to low copy numbers. To uncover the full regulatory network, it is essential to effectively recognize the functional epigenetic features and summarize the cell-type-specific patterns and interactions. Here, we used a multi-label deep neural network to learn the accessible patterns of each peak for every cell type. The model with a high prediction performance is the base model for further peak refinement and functional SNP identification.

**Figure 3:**
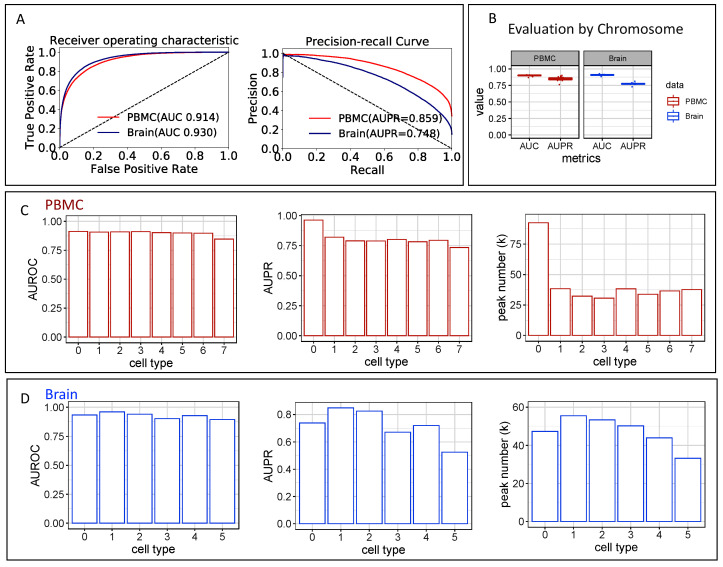
scEpiLock performance on the PBMC scATAC-seq data (**A**) ROC and PR curves for the PBMC and Brainsc ATAC-seq data. (**B**) Model performance on hold-out chromosomes. (**C**,**D**) AUC and AUPR and peak number counts for each cell type. (**C**) PBMC data, 0 to 7 represent CD14+ monocytes, CD4 memory cells, CD8 effector, CD4 naïve cells, CD8 naïve cells, pre-B cells, double negative T cells, and NK cells. (**D**) Brain data, 0 to 5 represent excitatory neurons, inhibitory neurons, microglia, oligodendrocytes, astrocytes, and OPCs.

We used two scATAC-seq datasets, PBMC and Brain, to evaluate the multi-label classifier’s performance in learning the patterns and predicting peak accessibility. We evaluated the model’s performance on both a randomly split test set and a hold-out chromosome. We benchmarked the scEpiLock model with previously developed deep learning models, DeepSEA and DanQ, and a Random Forest machine learning model. Consistently, we found that our scEpiLock model achieved the best performance in terms of both AUROC and AUPR (Table 1, Figure 3A). The first three deep learning models were trained with one NVIDIA GeForce RTX 3090 GPU, and the random forest model was trained with one Central Processing Unit (CPU). The processing time for each model can be found in Table S2. To further test the consistency of our model for all genomic regions, we performed leave-one-out cross-validation on all chromosomes and found that scEpiLock reported very similar performances among the chromosomes (Figure 3B). 

It is common to find unevenly cell-type-distributed peak numbers in scATAC-seq data. Due to uneven cell numbers, some cell types have many more called peaks than the other cell types. For instance, in the PBMC dataset, the CD14+ monocyte (cell type 0 in Figure 3C) was the dominant cell type. It had 1486 cells, while the other cell types had between 256 and 565 cells. The CD14+ monocyte also had more than double the number of peaks than the other cell types (93k vs. about 35k) (Figure 3C). We evaluated the model’s performance on each cell type separately to understand the impact of the peak number on the model’s performance. Despite the difference in peak numbers, all cell types had a similar AUROC. On the other hand, the CD14+ monocyte had an AUPR of 0.971, while the other cell types had AUPRs of between 0.749 and 0.830 (Figure 3C). This was as expected since the other cell types had many more negative labels than the CD14+ monocyte. Like PBMC, cell types in the brain have ab even AUROC, while AUPR is related to the peak number (Figure 3D).

### 3.2. Object Detection Module Precisely Refines Peak Boundry

The mean length of functional transcription factor binding sites for eukaryotes is 9.9 bp [15]. However, current scATAC-seq analysis methods give wide peaks with a length of around 500 bp [8]. That means the called peaks contain non-essential regions which disturb downstream analysis. Here, we refined the peak boundaries and detected key regions within the called peaks through the Grad-CAM object detection algorithm. 

**Figure 4:**
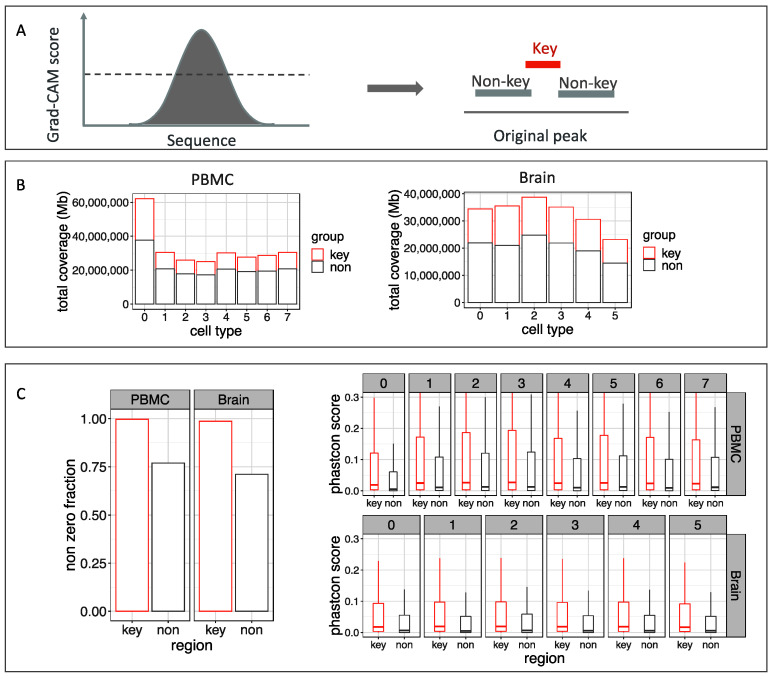
scEpiLock object detection module on the PBMC scATAC-seq data (**A**) Key regions with high Grad-CAM scores are selected. (**B**) Key region and non-key region coverage. (**C**) Nonzero PhastCons scores’ fractions between key and non-key regions; PhastCons scores’ comparison between key and non-key regions for each cell type.

The object detection model of scEpiLock is a weakly supervised localization that identifies the important regions in given peaks for predicting the label via Grad-CAM. It gives each base pair a Grad-CAM score representing its contribution to making predictions. A high Grad-CAM score corresponds to a higher importance placed on that position. We computed position-wise Grad-CAM scores on the PBMC scATAC-seq data and identified key regions composed of positions with Grad-CAM scores above the 80th percentile. This object detection module removed a significant proportion of the noise regions in the called peaks and condensed epigenomic features (Figure 4A). Grad-CAM reduced the peak size for every cell type on both datasets (Figure 4B). The total peak size was reduced to 1/3 of the original—261 Mb to 86 Mb for PBMC and 458 Mb to 161 Mb for the brain. 

To confirm that important regions were accurately selected by the object detection module, we compared the cross-species conservation scores of the excluded non-key regions vs. the selected key regions. Regions that serve important regulatory functions should be conserved, as any mutation would increase the likelihood of disadvantaged phenotypes [33]. Therefore, a higher conservation usually indicated important regions. We downloaded the 100-way PhastCons scores [35] and calculated the mean PhastCons score of each excluded non-key region and key region. We found all peaks for the PBMC PhastCons scores to be bigger than zero, while about 25% of the excluded non-ley regions have PhastCons scores of 0. We also compared the PhastCons scores’ distribution between the key and non-key regions for each cell type. We found all key regions have higher PhastCons scores than the non-key regions (*p*-value < 2.2 × 10^−16^, one-sided *t*-test) (Figure 4C. This demonstrated that the object detection module of scEpiLock refines the scATAC-seq peaks to the conserved and functional regions for each cell type.

To further validate that the selected key regions cover the regulatory and functional regions, e.g., enhancer, we compared the enrichment of H3K27ac between the key and non-key regions. H3K27ac is associated with the higher activation of transcription and is defined as an active enhancer marker [36]. We downloaded the bulk H3K27ac chromatin immunoprecipitation sequencing (ChIP-seq) bigwig files for PBMC and the brain. Then, we used the bigWigAverageOverBed function to compute the average score for big wig (H3K27ac enrichment) over the key and non-key regions. We found that for both the PBMC and brain data, the key regions have a higher average H3K27ac enrichment than the non-key regions. We performed a one-sided test and confirmed that H3K27ac was significantly enriched in the key regions compared to the non-key regions (Table S3). 

### 3.3. scEpiLock Predicts Functional Disease-Associated SNPs

Variants in the non-coding regions can play an important role in disease initiation and development [37]. The majority of the disease-causing mutations identified by GWAS lie within the non-coding regions such as enhancers, promoters, and insulators [38], and are covered by scATAC-seq. Thus, scATAC-seq data brings the opportunity to identify causal mutations for diseases and characterize their mechanisms of action. Here, we used scEpiLock to quantify the variant impact and impute the association between SNP and gene expression changes.

**Figure 5:**
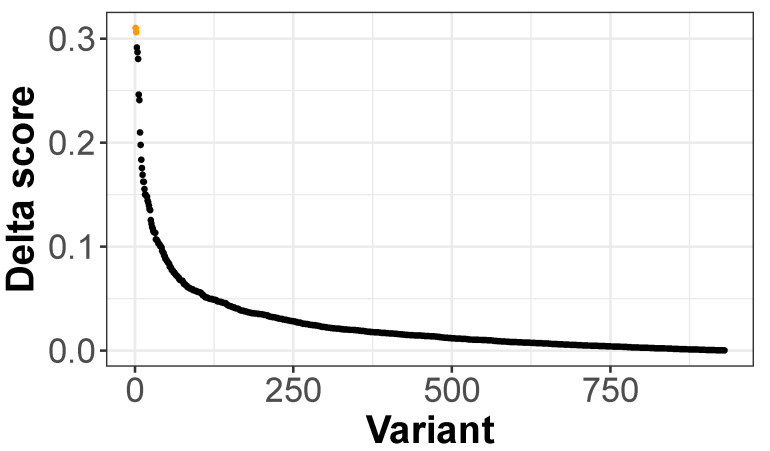
Delta scores of AD- and PD-related SNPS. rs1237999 and rs636317 are colored as orange.

We used the pre-trained scEpiLock model to calculate the accessible scores for both the WTs and mutants in the AD- and PD-related SNP sets [26]. The delta score was defined as the absolute difference of the accessible scores between the WT and mutants. High delta scores indicated that the SNP had a high probability of altering the peak accessibility. We ordered the 930 SNPs based on the computed delta scores and found rs12379999 and rs636317 had the highest delta scores (Figure 5). We discuss in detail below the model-identified top variant from the given SNPs list, but the scEpiLock model can be used to evaluate any given SNPs without retraining the model.

**Figure 6:**
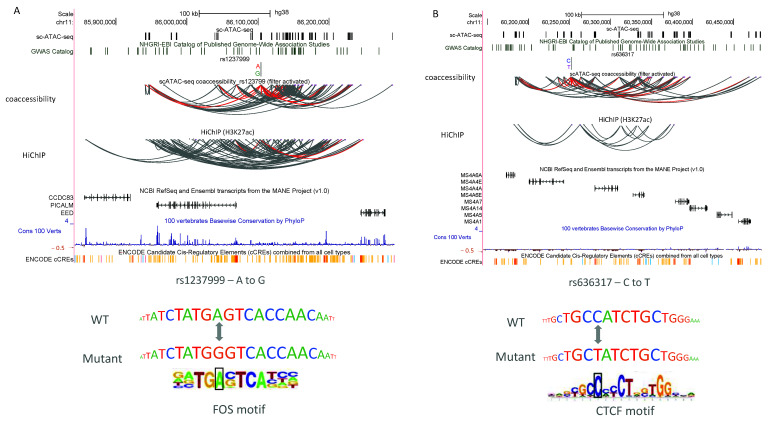
Visualization of scEpiLock-identified functional SNPs in AD and PD. (**A**,**B**) Reference genome, scATAC-seq peaks, GWAS SNPs, co-accessibility associations, H3K27ac HiChiP, nearby genes, Cons 100 Verts scores, ENCODE cCREs, and matching motifs for (**A**) rs1237999 (**B**) rs636317.

The scEpilock-identified important variants fell into two categories: (1) SNPs linked to established disease-related genes where the specific causative SNP is unknown; (2) SNPs linked to genes previously not implicated in disease etiology. We examined the top SNPs’ genomic locations, co-accessibility, 3D interactions with nearby genes, conservation scores, overlaps with ENCODE candidate *cis*-regulatory elements (cCREs), and motif patterns to reveal the mechanism. SNP rs12379999 had the highest delta score of 0.31. It is located around 37 kb upstream from the transcription starting site of PICALM, which is a well-known gene responsible for late-onset AD incidence [39]. In addition, it showed 3D interactions with both PICALM and EED, which is a polycomb-group family member maintaining a repressive transcriptional state. Lastly, it is located within one of the ENCODE cCREs regions and disturbs a putative FOS factor binding motif (Figure 6A). SNP rs12379999 has also been identified as a functional SNP by other methods [26] and is an example of how scEpiLock reveals the functional SNPs for both known and new disease-related genes. SNP rs636317 had the second highest delta score of 0.306. It is located upstream from several MS6A genes, which were previously reported as associating with AD by participating in the regulation of calcium signaling [40]. It had a co-accessibility with the MS6A gene clusters, and potentially impacted gene expression by disturbing the CTCF motif (Figure 6B). 

Besides the top two SNPs, we also examined other top SNPs in the list. For instance, rs10769263 decreased the accessible score from 0.723 to 0.485 for microglia, which indicated that this mutation converted the region from accessible to non-accessible. This SNP was located 17 kb upstream from the transcription start site of the SPI1 (PU.1) gene. In addition, the peaks containing the SNP and the transcription start site of SPI1 had a co-accessibility as shown in the co-accessibility track (Figure S4). This indicated that the SNP could have potentially impacted SPI1′s transcription. Previous research has shown that SPI1 regulates microglia development and function [41], and SPI1 expression changes impact AD progression [42,43]. However, not many studies focused on the function of non-coding variants. Our model predicted rs10769263 as a potential functional non-coding variation which communicated particularly with SPI1′s promoter, potentially by disrupting the Hoxc9 TF binding motif (Figure S4).

## 4. Discussion

We present scEpiLock, a weakly supervised deep learning framework to predict and refine chromatin accessible peaks and localize functional genomic variants in a cell-type-specific manner. Our model has three main modules: a multi-label classifier, an object detection module, and a variant impact quantification module. 

For the multi-label deep learning classifier, we trained a deep learning model on the chromatin accessible peaks of each cell type on scATAC-seq data. The CNN layers in the model have the capacity to learn genomic patterns and cell-type-specific regulatory interactions. The dense layers integrate the learned information and compute cell-type-specific accessible scores. We evaluated the model’s performance on two public datasets: PBMC scATAC-seq data and brain scATAC-seq dat. scEpiLock achieved state-of-the-art results on both datasets. With a pre-trained scEpiLock model, we can efficiently extract the complex gene regulatory patterns and predict the cell-type-specific accessibility for any given peaks if the cell type has been used to train the model. The model opens the door for low-cost personalized chromatin accessibility predictions—once trained on enough data, scEpilock can be used to predict the peak accessibility for multiple cell types, even though the peak sequence is patient-specific and has not been seen before. With more scATAC-seq datasets becoming available, we can expect scEpiLock to be used in many more different cell types.

In addition to multi-label predictions, the scEpiLock model also incorporates a boundary detection module via Grad-CAM. The position-wise importance scores can be used to refine the coarse peaks and identify the core functional regions. We showed that our condensed peaks have high PhastCons scores and are highly conserved during evolution. Moreover, scEpiLock has a variant impact quantification module to predict putative disease-associated SNPs. It calculates the accessible scores between WTs and mutants to identify SNPs that have a high impact on cell-type-specific chromatin accessibility.

There are several future directions to explore. One direction is to further improve the multi-label prediction module. For instance, bi-directional long short-term memory (bi-LSTM) layers can be incorporated to help capture interactions with long distances. Attention=based models can also be evaluated. On the other hand, Grad-CAM can be replaced by other object boundary detection methods, such as Grad-CAM++ [44], FullGrad [45], etc. We reason scEpiLock can serve as a base model to predict and refine scATAC-seq peak and evaluate functional SNPs, while the specific model structure or refinement method can be changed.

Taken together, we present a deep learning tool that could be widely deployed for cell-type-specific open chromatin identification. In addition, scEpiLock can refine the open chromatin peaks by boundary detection and predict disease-related SNPs. scEpilock is written as a Python package, and can easily be incorporated into existing acATAC-seq analysis pipelines, e.g., SnapATAC [7], ArchR [8].

## Figures and Tables

**Table 1:** Model performance comparison. scEpiLock has the best performance on both datasets.

Data	Model	AUROC	AUPR	F1 Score
**PBMC**	**scEpiLock**	**0.914**	**0.854**	**0.752**
	DeepSEA	0.906	0.841	0.742
	DanQ	0.905	0.841	0.753
	RF	0.79	0.681	0.451
**Brain**	**scEpiLock**	**0.93**	**0.745**	**0.669**
	DeepSEA	0.92	0.72	0.634
	DanQ	0.92	0.717	0.642
	RF	0.576	0.249	0

## Data Availability

Data and codes can be found at https://github.com/aicb-ZhangLabs/scEpiLock (accessed on 16 June 2022).

## Supplementary Materials

The following supporting information can be downloaded at: https://www.mdpi.com/article/10.3390/biom12070874/s1, Figure S1: Positive label distribution for each peak of PBMC; Figure S2: Positive label distribution for each peak of the brain data; Figure S3: Transfer learning improves multi-label classifier’s performance on small scATAC-seq dataset; Figure S4: Visualization of scEpiLock-identified functional SNP rs10769263; Table S1: ENCODE Bulk ATAC-seq Sample List for Transfer Learning; Table S2 Computing Power and Processing Time for Each Model; Table S3 H3K27ac Enrichment in Key and Non-key Regions; Note S1: Detailed specifications of the architectures and hyperparameters of scEpiLock multi-label classifier module.
