# Supplementary material for: scEpiLock: A Weakly Supervised Learning Framework for cis-Regulatory Element Localization and Variant Impact Quantification for Single-Cell Epigenetic Data
Source: Biomolecules. 2022 Jun 23;12(7):874. doi: 10.3390/biom12070874 (PMC9312957; doi:10.3390/biom12070874)
Supplement: Supplementary file 1 [file biomolecules-12-00874-s001.zip › biomolecules-1736338-supplementary.pdf]

Supplementary Notes for scEpiLock: A Weakly-Supervised Learning Framework for cis-Regulatory Element Localization and Variant Impact Quantification for Single-Cell Epigenetic Data

Yanwen Gong[1,2], Shushrruth Sai Srinivasan[3], Ruiyi Zhang[3], Kai Kessenbrock[1,2,*] and Jing Zhang[3,*]

[1]Center for Complex Biological Systems, University of California, Irvine, CA 92697, USA
[2]Department of Biological Chemistry, School of Medicine, University of California, Irvine, CA 92697, USA
[3]Department of Computer Science, University of California, Irvine, CA 92697, USA

*To whom correspondence should be addressed.

Table of Contents

Note S1 Detailed specifications of the architectures and hyperparameters of scEpiLock multi-label classifier module 3

Supplementary Figures 4

Figure S1 Positive label distribution for each peak of PBMC 4

Figure S2 Positive label distribution for each peak of the brain data 5

Supplementary Figure 3 Transfer learning improves multi-label classifier's performance on small scATAC-seq dataset 6

Figure S4 Visualization of scEpiLock identified functional SNP rs10769263 7

Table S1 ENCODE Bulk ATAC-seq Sample List for Transfer Learning 8

Table S2 Computing Power and Processing Time for Each Model 21

Table S3 H3K27ac Enrichment in Key and Non-key Regions 22

Note S1 Detailed specifications of the architectures and hyperparameters of scEpiLock multi-label classifier module

scEpiLock uses a multilayer CNN model, which contains four convolutional layers, two pooling layers and two dense fully connected layers. The filters in the convolutional layers are trained to recognize the CREs. The dense fully connected dense layers combine the learnt information and output the cell-type specific peak accessible score. The model is organized into a sequential layer-by-layer structure with each layer as a functional transformation.

Model Architecture:

1. Convolution layer (320 channels, kernel size 8, stride 1)
2. Convolution layer (480 channels, kernel size 8, stride 1)
3. Max pooling layer (kernel size 4, stride 4)
4. Convolution layer (960 channels, kernel size 4, stride 1)
5. Max pooling layer (kernel size 4, stride 4)
6. Convolution layer (1024 channels, kernel size 4, stride 1)
7. Fully connected layer1
8. Fully connected layer2

To avoid overfitting, we added dropouts to randomly set a proportion of neuron activations to a value of 0.

Dropout proportion (proportion of outputs randomly set to 0):

- Layer3: 20%
- Layer5: 50%
- Layer6: 50%
- Other layers: 0%

Supplementary Figures

Figure S1 Positive label distribution for each peak of PBMC

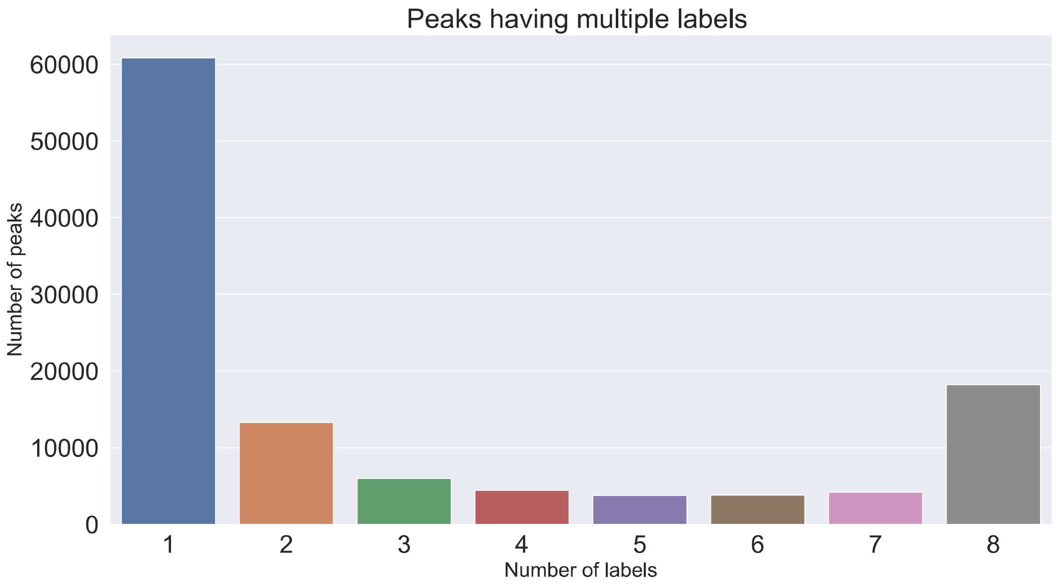

In the PBMC scATAC-seq data, 53.1% of peaks (n = 60,834 peaks) are unique to one cell type while 15.9% of peaks (n = 18,209 peaks) are shared among the eight cell types. Rest of the peaks are shared among two to seven cell types

Figure S2 Positive label distribution for each peak of the brain data

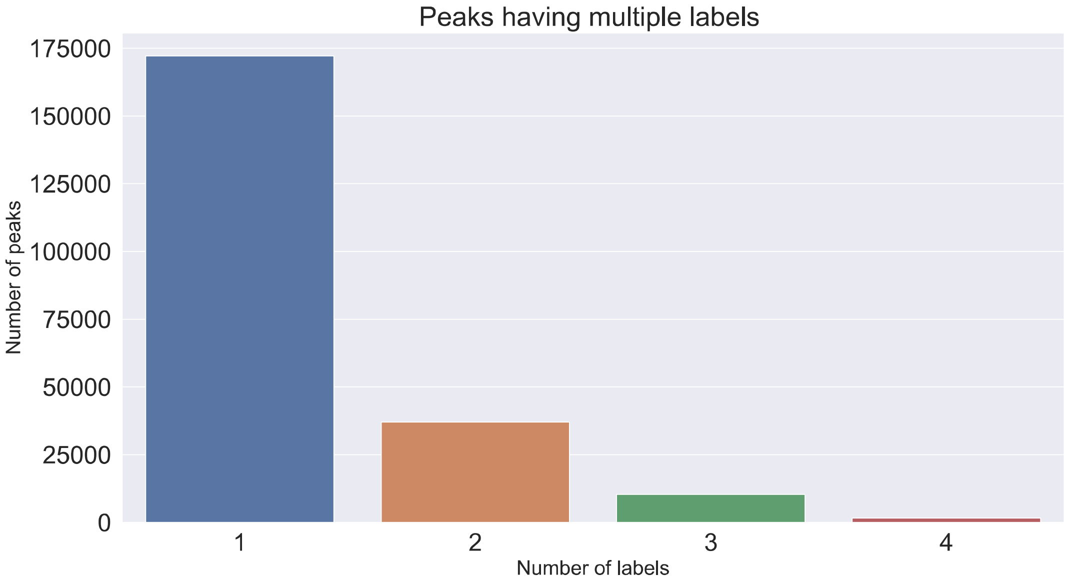

In the brain scATAC-seq data, 77.9% (n = 172,111 peaks) of peaks are specific to a single cell type and the rest of peaks are shared among at least two cell types

Supplementary Figure 3 Transfer learning improves multi-label classifier's performance on small scATAC-seq dataset

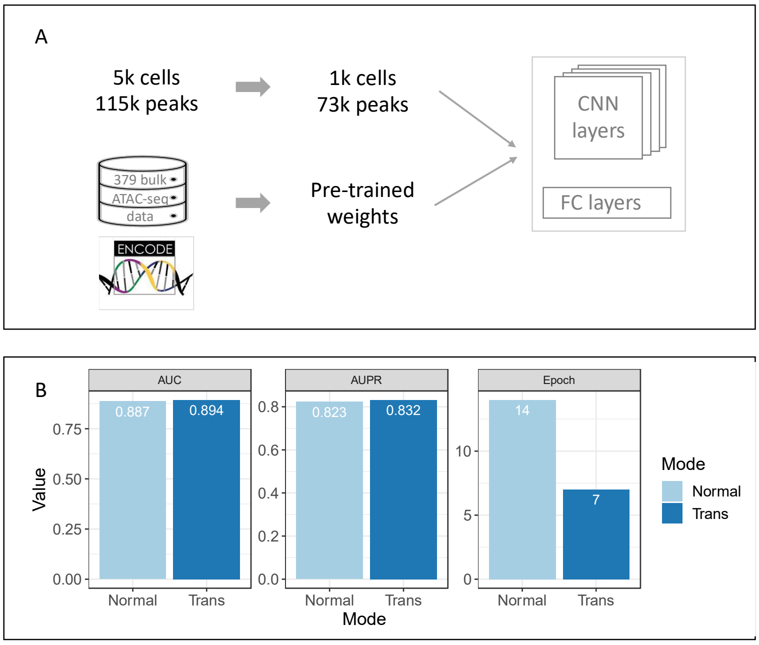

(A) ENCODE bulk ATAC-seq data was used to generate pre-trained weights of the classifier. The 5k PBMC data was filtered to only include 1k cells with 73k peaks representing a small scATAC-seq dataset. The pre-trained weights and subset dataset were fed into the multi-label classifier. (B) Transfer learning model achieved higher AUC and AUPR than model with random initiated weights. In addition, transfer learning reduced the training time to half of before.

Figure S4 Visualization of scEpiLock identified functional SNP rs10769263

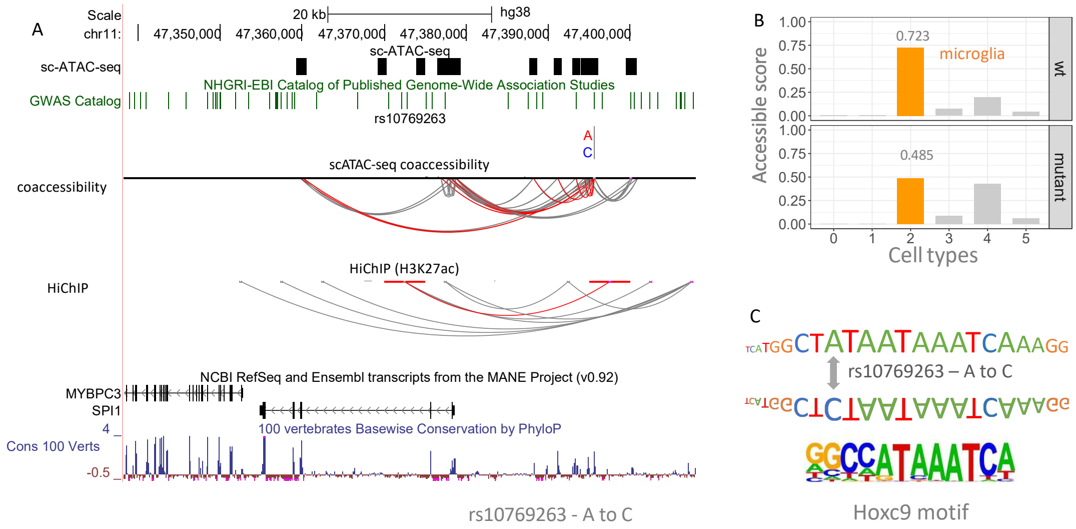

(A) Brain scATAC-seq pseudobulk, GWAS, rs10769263, co-accessibility, HiChIP, nearby genes, PhastCons 100 vertebrates track on UCSC genome browser. (B) Predicted accessible scores of WT and mutant. (C) Sequences contain rs10759263 SNP aligned with Hoxc9 motif logo

Table S1 ENCODE Bulk ATAC-seq Sample List for Transfer Learning

File.accession	Experiment.accession	File.download.URL
ENCFF063AGI	ENCSR554WGQ	https://www.encodeproject.org/files/ENCFF063AGI/@@download/ENCFF063AGI.bed.gz
ENCFF965AOW	ENCSR383RDZ	https://www.encodeproject.org/files/ENCFF965AOW/@@download/ENCFF965AOW.bed.gz
ENCFF007FSO	ENCSR684KUG	https://www.encodeproject.org/files/ENCFF007FSO/@@download/ENCFF007FSO.bed.gz
ENCFF132ATE	ENCSR368FYV	https://www.encodeproject.org/files/ENCFF132ATE/@@download/ENCFF132ATE.bed.gz
ENCFF110LOX	ENCSR220ASC	https://www.encodeproject.org/files/ENCFF110LOX/@@download/ENCFF110LOX.bed.gz
ENCFF008FUL	ENCSR220ASC	https://www.encodeproject.org/files/ENCFF008FUL/@@download/ENCFF008FUL.bed.gz
ENCFF181FFW	ENCSR220ASC	https://www.encodeproject.org/files/ENCFF181FFW/@@download/ENCFF181FFW.bed.gz
ENCFF463DJH	ENCSR220ASC	https://www.encodeproject.org/files/ENCFF463DJH/@@download/ENCFF463DJH.bed.gz
ENCFF172ZTK	ENCSR220ASC	https://www.encodeproject.org/files/ENCFF172ZTK/@@download/ENCFF172ZTK.bed.gz
ENCFF795ASF	ENCSR220ASC	https://www.encodeproject.org/files/ENCFF795ASF/@@download/ENCFF795ASF.bed.gz
ENCFF516KWQ	ENCSR220ASC	https://www.encodeproject.org/files/ENCFF516KWQ/@@download/ENCFF516KWQ.bed.gz
ENCFF744XPE	ENCSR824DUE	https://www.encodeproject.org/files/ENCFF744XPE/@@download/ENCFF744XPE.bed.gz
ENCFF453BWQ	ENCSR673ZMQ	https://www.encodeproject.org/files/ENCFF453BWQ/@@download/ENCFF453BWQ.bed.gz
ENCFF857VFO	ENCSR180HEL	https://www.encodeproject.org/files/ENCFF857VFO/@@download/ENCFF857VFO.bed.gz
ENCFF766BZE	ENCSR512YXO	https://www.encodeproject.org/files/ENCFF766BZE/@@download/ENCFF766BZE.bed.gz
ENCFF796GGQ	ENCSR249FXU	https://www.encodeproject.org/files/ENCFF796GGQ/@@download/ENCFF796GGQ.bed.gz
ENCFF411CDL	ENCSR939EVW	https://www.encodeproject.org/files/ENCFF411CDL/@@download/ENCFF411CDL.bed.gz
ENCFF785YXH	ENCSR105SCQ	https://www.encodeproject.org/files/ENCFF785YXH/@@download/ENCFF785YXH.bed.gz
ENCFF916HWL	ENCSR381LJX	https://www.encodeproject.org/files/ENCFF916HWL/@@download/ENCFF916HWL.bed.gz
ENCFF489VFL	ENCSR940YDN	https://www.encodeproject.org/files/ENCFF489VFL/@@download/ENCFF489VFL.bed.gz
ENCFF209BNR	ENCSR372IGW	https://www.encodeproject.org/files/ENCFF209BNR/@@download/ENCFF209BNR.bed.gz
ENCFF816FSQ	ENCSR025MEO	https://www.encodeproject.org/files/ENCFF816FSQ/@@download/ENCFF816FSQ.bed.gz
ENCFF630QOE	ENCSR400ISH	https://www.encodeproject.org/files/ENCFF630QOE/@@download/ENCFF630QOE.bed.gz
ENCFF796ZYH	ENCSR419UYY	https://www.encodeproject.org/files/ENCFF796ZYH/@@download/ENCFF796ZYH.bed.gz

ENCFF728CSD	ENCSR712ZRY	https://www.encodeproject.org/files/ENCFF728CSD/@@download/ENCFF728CSD.bed.gz
ENCFF816FTJ	ENCSR608KJD	https://www.encodeproject.org/files/ENCFF816FTJ/@@download/ENCFF816FTJ.bed.gz
ENCFF624KRL	ENCSR905PWV	https://www.encodeproject.org/files/ENCFF624KRL/@@download/ENCFF624KRL.bed.gz
ENCFF044HAG	ENCSR315QWI	https://www.encodeproject.org/files/ENCFF044HAG/@@download/ENCFF044HAG.bed.gz
ENCFF976AUE	ENCSR604YEQ	https://www.encodeproject.org/files/ENCFF976AUE/@@download/ENCFF976AUE.bed.gz
ENCFF961CHG	ENCSR312UCH	https://www.encodeproject.org/files/ENCFF961CHG/@@download/ENCFF961CHG.bed.gz
ENCFF136RJT	ENCSR189VFC	https://www.encodeproject.org/files/ENCFF136RJT/@@download/ENCFF136RJT.bed.gz
ENCFF305ZQQ	ENCSR278JWM	https://www.encodeproject.org/files/ENCFF305ZQQ/@@download/ENCFF305ZQQ.bed.gz
ENCFF374TYJ	ENCSR839UOB	https://www.encodeproject.org/files/ENCFF374TYJ/@@download/ENCFF374TYJ.bed.gz
ENCFF780EDM	ENCSR420RVW	https://www.encodeproject.org/files/ENCFF780EDM/@@download/ENCFF780EDM.bed.gz
ENCFF761RRO	ENCSR615YIL	https://www.encodeproject.org/files/ENCFF761RRO/@@download/ENCFF761RRO.bed.gz
ENCFF182SEJ	ENCSR954BDA	https://www.encodeproject.org/files/ENCFF182SEJ/@@download/ENCFF182SEJ.bed.gz
ENCFF505YPT	ENCSR163KRG	https://www.encodeproject.org/files/ENCFF505YPT/@@download/ENCFF505YPT.bed.gz
ENCFF551EAY	ENCSR637MUF	https://www.encodeproject.org/files/ENCFF551EAY/@@download/ENCFF551EAY.bed.gz
ENCFF550WFX	ENCSR635AIF	https://www.encodeproject.org/files/ENCFF550WFX/@@download/ENCFF550WFX.bed.gz
ENCFF252MOE	ENCSR594TMY	https://www.encodeproject.org/files/ENCFF252MOE/@@download/ENCFF252MOE.bed.gz
ENCFF810XER	ENCSR040PBN	https://www.encodeproject.org/files/ENCFF810XER/@@download/ENCFF810XER.bed.gz
ENCFF471QJG	ENCSR833ACP	https://www.encodeproject.org/files/ENCFF471QJG/@@download/ENCFF471QJG.bed.gz
ENCFF246XGY	ENCSR000RBT	https://www.encodeproject.org/files/ENCFF246XGY/@@download/ENCFF246XGY.bed.gz
ENCFF508EYC	ENCSR528YDD	https://www.encodeproject.org/files/ENCFF508EYC/@@download/ENCFF508EYC.bed.gz
ENCFF176CKB	ENCSR231BZU	https://www.encodeproject.org/files/ENCFF176CKB/@@download/ENCFF176CKB.bed.gz
ENCFF209RWV	ENCSR024WOD	https://www.encodeproject.org/files/ENCFF209RWV/@@download/ENCFF209RWV.bed.gz
ENCFF715VOA	ENCSR524CPZ	https://www.encodeproject.org/files/ENCFF715VOA/@@download/ENCFF715VOA.bed.gz
ENCFF468CHZ	ENCSR470ZNM	https://www.encodeproject.org/files/ENCFF468CHZ/@@download/ENCFF468CHZ.bed.gz
ENCFF594MCK	ENCSR924SDV	https://www.encodeproject.org/files/ENCFF594MCK/@@download/ENCFF594MCK.bed.gz
ENCFF610BYY	ENCSR628PLS	https://www.encodeproject.org/files/ENCFF610BYY/@@download/ENCFF610BYY.bed.gz
ENCFF129JFU	ENCSR233HSC	https://www.encodeproject.org/files/ENCFF129JFU/@@download/ENCFF129JFU.bed.gz
ENCFF197NOP	ENCSR425WQW	https://www.encodeproject.org/files/ENCFF197NOP/@@download/ENCFF197NOP.bed.gz
ENCFF437HEK	ENCSR821QHC	https://www.encodeproject.org/files/ENCFF437HEK/@@download/ENCFF437HEK.bed.gz
ENCFF138FQA	ENCSR335JVB	https://www.encodeproject.org/files/ENCFF138FQA/@@download/ENCFF138FQA.bed.gz
ENCFF876LVG	ENCSR960KGO	https://www.encodeproject.org/files/ENCFF876LVG/@@download/ENCFF876LVG.bed.gz

ENCFF344XDS	ENCSR917WJS	https://www.encodeproject.org/files/ENCFF344XDS/@@download/ENCFF344XDS.bed.gz
ENCFF340FLI	ENCSR854ZBA	https://www.encodeproject.org/files/ENCFF340FLI/@@download/ENCFF340FLI.bed.gz
ENCFF452XUT	ENCSR700HPA	https://www.encodeproject.org/files/ENCFF452XUT/@@download/ENCFF452XUT.bed.gz
ENCFF002SXG	ENCSR134OSR	https://www.encodeproject.org/files/ENCFF002SXG/@@download/ENCFF002SXG.bed.gz
ENCFF844BQZ	ENCSR424KQH	https://www.encodeproject.org/files/ENCFF844BQZ/@@download/ENCFF844BQZ.bed.gz
ENCFF918IOX	ENCSR123WME	https://www.encodeproject.org/files/ENCFF918IOX/@@download/ENCFF918IOX.bed.gz
ENCFF762FXN	ENCSR096VEO	https://www.encodeproject.org/files/ENCFF762FXN/@@download/ENCFF762FXN.bed.gz
ENCFF682ZZT	ENCSR359HFH	https://www.encodeproject.org/files/ENCFF682ZZT/@@download/ENCFF682ZZT.bed.gz
ENCFF756WUH	ENCSR821JHD	https://www.encodeproject.org/files/ENCFF756WUH/@@download/ENCFF756WUH.bed.gz
ENCFF107CMG	ENCSR376YMU	https://www.encodeproject.org/files/ENCFF107CMG/@@download/ENCFF107CMG.bed.gz
ENCFF733DJY	ENCSR084FHK	https://www.encodeproject.org/files/ENCFF733DJY/@@download/ENCFF733DJY.bed.gz
ENCFF937GVS	ENCSR305XRF	https://www.encodeproject.org/files/ENCFF937GVS/@@download/ENCFF937GVS.bed.gz
ENCFF093GBP	ENCSR635CAC	https://www.encodeproject.org/files/ENCFF093GBP/@@download/ENCFF093GBP.bed.gz
ENCFF339RRA	ENCSR920FQJ	https://www.encodeproject.org/files/ENCFF339RRA/@@download/ENCFF339RRA.bed.gz
ENCFF694FCS	ENCSR762YFD	https://www.encodeproject.org/files/ENCFF694FCS/@@download/ENCFF694FCS.bed.gz
ENCFF061UDU	ENCSR275LCF	https://www.encodeproject.org/files/ENCFF061UDU/@@download/ENCFF061UDU.bed.gz
ENCFF703AAZ	ENCSR422RPD	https://www.encodeproject.org/files/ENCFF703AAZ/@@download/ENCFF703AAZ.bed.gz
ENCFF262DJL	ENCSR208HAP	https://www.encodeproject.org/files/ENCFF262DJL/@@download/ENCFF262DJL.bed.gz
ENCFF278HDB	ENCSR011NJP	https://www.encodeproject.org/files/ENCFF278HDB/@@download/ENCFF278HDB.bed.gz
ENCFF966KNT	ENCSR487QSB	https://www.encodeproject.org/files/ENCFF966KNT/@@download/ENCFF966KNT.bed.gz
ENCFF367ROZ	ENCSR038WVV	https://www.encodeproject.org/files/ENCFF367ROZ/@@download/ENCFF367ROZ.bed.gz
ENCFF674XMD	ENCSR214EIV	https://www.encodeproject.org/files/ENCFF674XMD/@@download/ENCFF674XMD.bed.gz
ENCFF085DFN	ENCSR357WQH	https://www.encodeproject.org/files/ENCFF085DFN/@@download/ENCFF085DFN.bed.gz
ENCFF586OGP	ENCSR442OAV	https://www.encodeproject.org/files/ENCFF586OGP/@@download/ENCFF586OGP.bed.gz
ENCFF557BSF	ENCSR304ZDQ	https://www.encodeproject.org/files/ENCFF557BSF/@@download/ENCFF557BSF.bed.gz
ENCFF384IUM	ENCSR122NDR	https://www.encodeproject.org/files/ENCFF384IUM/@@download/ENCFF384IUM.bed.gz
ENCFF464KLS	ENCSR404OEO	https://www.encodeproject.org/files/ENCFF464KLS/@@download/ENCFF464KLS.bed.gz
ENCFF030FZS	ENCSR408IVU	https://www.encodeproject.org/files/ENCFF030FZS/@@download/ENCFF030FZS.bed.gz
ENCFF256UDE	ENCSR857MCZ	https://www.encodeproject.org/files/ENCFF256UDE/@@download/ENCFF256UDE.bed.gz
ENCFF948GVD	ENCSR835WBW	https://www.encodeproject.org/files/ENCFF948GVD/@@download/ENCFF948GVD.bed.gz
ENCFF268EVB	ENCSR962ITF	https://www.encodeproject.org/files/ENCFF268EVB/@@download/ENCFF268EVB.bed.gz

ENCFF695HXA	ENCSR253QLW	https://www.encodeproject.org/files/ENCFF695HXA/@@download/ENCFF695HXA.bed.gz
ENCFF538YUQ	ENCSR492FJO	https://www.encodeproject.org/files/ENCFF538YUQ/@@download/ENCFF538YUQ.bed.gz
ENCFF451ZYJ	ENCSR858GTE	https://www.encodeproject.org/files/ENCFF451ZYJ/@@download/ENCFF451ZYJ.bed.gz
ENCFF619LDY	ENCSR322SBN	https://www.encodeproject.org/files/ENCFF619LDY/@@download/ENCFF619LDY.bed.gz
ENCFF356XWJ	ENCSR621JYI	https://www.encodeproject.org/files/ENCFF356XWJ/@@download/ENCFF356XWJ.bed.gz
ENCFF423TQD	ENCSR512FHU	https://www.encodeproject.org/files/ENCFF423TQD/@@download/ENCFF423TQD.bed.gz
ENCFF597KPI	ENCSR839IAD	https://www.encodeproject.org/files/ENCFF597KPI/@@download/ENCFF597KPI.bed.gz
ENCFF184NOC	ENCSR509JPT	https://www.encodeproject.org/files/ENCFF184NOC/@@download/ENCFF184NOC.bed.gz
ENCFF390QUM	ENCSR487SOP	https://www.encodeproject.org/files/ENCFF390QUM/@@download/ENCFF390QUM.bed.gz
ENCFF926ULQ	ENCSR706GDQ	https://www.encodeproject.org/files/ENCFF926ULQ/@@download/ENCFF926ULQ.bed.gz
ENCFF697QXK	ENCSR144ZXR	https://www.encodeproject.org/files/ENCFF697QXK/@@download/ENCFF697QXK.bed.gz
ENCFF003NCK	ENCSR409YCK	https://www.encodeproject.org/files/ENCFF003NCK/@@download/ENCFF003NCK.bed.gz
ENCFF611XGS	ENCSR454UTH	https://www.encodeproject.org/files/ENCFF611XGS/@@download/ENCFF611XGS.bed.gz
ENCFF962LEY	ENCSR063UNG	https://www.encodeproject.org/files/ENCFF962LEY/@@download/ENCFF962LEY.bed.gz
ENCFF598EDX	ENCSR624CTU	https://www.encodeproject.org/files/ENCFF598EDX/@@download/ENCFF598EDX.bed.gz
ENCFF007OSO	ENCSR345NVR	https://www.encodeproject.org/files/ENCFF007OSO/@@download/ENCFF007OSO.bed.gz
ENCFF900WSD	ENCSR902CFR	https://www.encodeproject.org/files/ENCFF900WSD/@@download/ENCFF900WSD.bed.gz
ENCFF072JKR	ENCSR241VGH	https://www.encodeproject.org/files/ENCFF072JKR/@@download/ENCFF072JKR.bed.gz
ENCFF027KMZ	ENCSR331JFZ	https://www.encodeproject.org/files/ENCFF027KMZ/@@download/ENCFF027KMZ.bed.gz
ENCFF915GOP	ENCSR580JBA	https://www.encodeproject.org/files/ENCFF915GOP/@@download/ENCFF915GOP.bed.gz
ENCFF828UQL	ENCSR641KNU	https://www.encodeproject.org/files/ENCFF828UQL/@@download/ENCFF828UQL.bed.gz
ENCFF228KGP	ENCSR553LAZ	https://www.encodeproject.org/files/ENCFF228KGP/@@download/ENCFF228KGP.bed.gz
ENCFF316GRQ	ENCSR775SYU	https://www.encodeproject.org/files/ENCFF316GRQ/@@download/ENCFF316GRQ.bed.gz
ENCFF245FHK	ENCSR344ZTM	https://www.encodeproject.org/files/ENCFF245FHK/@@download/ENCFF245FHK.bed.gz
ENCFF940NBD	ENCSR685ZMP	https://www.encodeproject.org/files/ENCFF940NBD/@@download/ENCFF940NBD.bed.gz
ENCFF423RGJ	ENCSR356KRQ	https://www.encodeproject.org/files/ENCFF423RGJ/@@download/ENCFF423RGJ.bed.gz
ENCFF768TYT	ENCSR356KRQ	https://www.encodeproject.org/files/ENCFF768TYT/@@download/ENCFF768TYT.bed.gz
ENCFF236BWF	ENCSR356KRQ	https://www.encodeproject.org/files/ENCFF236BWF/@@download/ENCFF236BWF.bed.gz
ENCFF287WFV	ENCSR356KRQ	https://www.encodeproject.org/files/ENCFF287WFV/@@download/ENCFF287WFV.bed.gz
ENCFF616CHX	ENCSR552QUA	https://www.encodeproject.org/files/ENCFF616CHX/@@download/ENCFF616CHX.bed.gz
ENCFF767OIB	ENCSR990NNX	https://www.encodeproject.org/files/ENCFF767OIB/@@download/ENCFF767OIB.bed.gz

ENCFF324QLD	ENCSR496PPU	https://www.encodeproject.org/files/ENCFF324QLD/@@download/ENCFF324QLD.bed.gz
ENCFF483FWG	ENCSR836FIL	https://www.encodeproject.org/files/ENCFF483FWG/@@download/ENCFF483FWG.bed.gz
ENCFF820DKN	ENCSR452OSK	https://www.encodeproject.org/files/ENCFF820DKN/@@download/ENCFF820DKN.bed.gz
ENCFF976AWF	ENCSR624ODL	https://www.encodeproject.org/files/ENCFF976AWF/@@download/ENCFF976AWF.bed.gz
ENCFF695NDC	ENCSR133WJY	https://www.encodeproject.org/files/ENCFF695NDC/@@download/ENCFF695NDC.bed.gz
ENCFF829YTT	ENCSR251POP	https://www.encodeproject.org/files/ENCFF829YTT/@@download/ENCFF829YTT.bed.gz
ENCFF776LMY	ENCSR474XFV	https://www.encodeproject.org/files/ENCFF776LMY/@@download/ENCFF776LMY.bed.gz
ENCFF365RAW	ENCSR474XFV	https://www.encodeproject.org/files/ENCFF365RAW/@@download/ENCFF365RAW.bed.gz
ENCFF826MFY	ENCSR414DVK	https://www.encodeproject.org/files/ENCFF826MFY/@@download/ENCFF826MFY.bed.gz
ENCFF663KQH	ENCSR414DVK	https://www.encodeproject.org/files/ENCFF663KQH/@@download/ENCFF663KQH.bed.gz
ENCFF410GKB	ENCSR668VCT	https://www.encodeproject.org/files/ENCFF410GKB/@@download/ENCFF410GKB.bed.gz
ENCFF251CFX	ENCSR668VCT	https://www.encodeproject.org/files/ENCFF251CFX/@@download/ENCFF251CFX.bed.gz
ENCFF541ZHB	ENCSR355SGJ	https://www.encodeproject.org/files/ENCFF541ZHB/@@download/ENCFF541ZHB.bed.gz
ENCFF323ALI	ENCSR355SGJ	https://www.encodeproject.org/files/ENCFF323ALI/@@download/ENCFF323ALI.bed.gz
ENCFF117MSK	ENCSR483RKN	https://www.encodeproject.org/files/ENCFF117MSK/@@download/ENCFF117MSK.bed.gz
ENCFF695IGF	ENCSR483RKN	https://www.encodeproject.org/files/ENCFF695IGF/@@download/ENCFF695IGF.bed.gz
ENCFF976CEI	ENCSR483RKN	https://www.encodeproject.org/files/ENCFF976CEI/@@download/ENCFF976CEI.bed.gz
ENCFF925CYR	ENCSR483RKN	https://www.encodeproject.org/files/ENCFF925CYR/@@download/ENCFF925CYR.bed.gz
ENCFF932OLP	ENCSR392UJM	https://www.encodeproject.org/files/ENCFF932OLP/@@download/ENCFF932OLP.bed.gz
ENCFF389WIM	ENCSR886VCC	https://www.encodeproject.org/files/ENCFF389WIM/@@download/ENCFF389WIM.bed.gz
ENCFF800IWT	ENCSR886VCC	https://www.encodeproject.org/files/ENCFF800IWT/@@download/ENCFF800IWT.bed.gz
ENCFF105YML	ENCSR113MBR	https://www.encodeproject.org/files/ENCFF105YML/@@download/ENCFF105YML.bed.gz
ENCFF460SWB	ENCSR113MBR	https://www.encodeproject.org/files/ENCFF460SWB/@@download/ENCFF460SWB.bed.gz
ENCFF948OYI	ENCSR120MOY	https://www.encodeproject.org/files/ENCFF948OYI/@@download/ENCFF948OYI.bed.gz
ENCFF168ASJ	ENCSR120MOY	https://www.encodeproject.org/files/ENCFF168ASJ/@@download/ENCFF168ASJ.bed.gz
ENCFF384SZZ	ENCSR890DWH	https://www.encodeproject.org/files/ENCFF384SZZ/@@download/ENCFF384SZZ.bed.gz
ENCFF775KEG	ENCSR890DWH	https://www.encodeproject.org/files/ENCFF775KEG/@@download/ENCFF775KEG.bed.gz
ENCFF682IJY	ENCSR952SPO	https://www.encodeproject.org/files/ENCFF682IJY/@@download/ENCFF682IJY.bed.gz
ENCFF981WQJ	ENCSR530XBF	https://www.encodeproject.org/files/ENCFF981WQJ/@@download/ENCFF981WQJ.bed.gz
ENCFF122OHC	ENCSR399OSE	https://www.encodeproject.org/files/ENCFF122OHC/@@download/ENCFF122OHC.bed.gz
ENCFF718BTP	ENCSR399OSE	https://www.encodeproject.org/files/ENCFF718BTP/@@download/ENCFF718BTP.bed.gz

ENCFF859RAO	ENCSR731ODJ	https://www.encodeproject.org/files/ENCFF859RAO/@@download/ENCFF859RAO.bed.gz
ENCFF991MKU	ENCSR731ODJ	https://www.encodeproject.org/files/ENCFF991MKU/@@download/ENCFF991MKU.bed.gz
ENCFF647AGG	ENCSR872WGW	https://www.encodeproject.org/files/ENCFF647AGG/@@download/ENCFF647AGG.bed.gz
ENCFF429LWF	ENCSR872WGW	https://www.encodeproject.org/files/ENCFF429LWF/@@download/ENCFF429LWF.bed.gz
ENCFF054UBO	ENCSR872WGW	https://www.encodeproject.org/files/ENCFF054UBO/@@download/ENCFF054UBO.bed.gz
ENCFF070LSQ	ENCSR872WGW	https://www.encodeproject.org/files/ENCFF070LSQ/@@download/ENCFF070LSQ.bed.gz
ENCFF986AJX	ENCSR855BMI	https://www.encodeproject.org/files/ENCFF986AJX/@@download/ENCFF986AJX.bed.gz
ENCFF363FTI	ENCSR855BMI	https://www.encodeproject.org/files/ENCFF363FTI/@@download/ENCFF363FTI.bed.gz
ENCFF009FWX	ENCSR212LYK	https://www.encodeproject.org/files/ENCFF009FWX/@@download/ENCFF009FWX.bed.gz
ENCFF968YWL	ENCSR212LYK	https://www.encodeproject.org/files/ENCFF968YWL/@@download/ENCFF968YWL.bed.gz
ENCFF643QLU	ENCSR439TZT	https://www.encodeproject.org/files/ENCFF643QLU/@@download/ENCFF643QLU.bed.gz
ENCFF645EFP	ENCSR925LGW	https://www.encodeproject.org/files/ENCFF645EFP/@@download/ENCFF645EFP.bed.gz
ENCFF984UBD	ENCSR925LGW	https://www.encodeproject.org/files/ENCFF984UBD/@@download/ENCFF984UBD.bed.gz
ENCFF901IVP	ENCSR609GST	https://www.encodeproject.org/files/ENCFF901IVP/@@download/ENCFF901IVP.bed.gz
ENCFF272UWG	ENCSR609GST	https://www.encodeproject.org/files/ENCFF272UWG/@@download/ENCFF272UWG.bed.gz
ENCFF072OBU	ENCSR548KIL	https://www.encodeproject.org/files/ENCFF072OBU/@@download/ENCFF072OBU.bed.gz
ENCFF511DPE	ENCSR548KIL	https://www.encodeproject.org/files/ENCFF511DPE/@@download/ENCFF511DPE.bed.gz
ENCFF834ZVG	ENCSR227FVE	https://www.encodeproject.org/files/ENCFF834ZVG/@@download/ENCFF834ZVG.bed.gz
ENCFF628CCO	ENCSR227FVE	https://www.encodeproject.org/files/ENCFF628CCO/@@download/ENCFF628CCO.bed.gz
ENCFF440HSL	ENCSR086OGH	https://www.encodeproject.org/files/ENCFF440HSL/@@download/ENCFF440HSL.bed.gz
ENCFF697MUB	ENCSR086OGH	https://www.encodeproject.org/files/ENCFF697MUB/@@download/ENCFF697MUB.bed.gz
ENCFF129BFZ	ENCSR078EBD	https://www.encodeproject.org/files/ENCFF129BFZ/@@download/ENCFF129BFZ.bed.gz
ENCFF105FRE	ENCSR078EBD	https://www.encodeproject.org/files/ENCFF105FRE/@@download/ENCFF105FRE.bed.gz
ENCFF250CRB	ENCSR851EBF	https://www.encodeproject.org/files/ENCFF250CRB/@@download/ENCFF250CRB.bed.gz
ENCFF560EBG	ENCSR851EBF	https://www.encodeproject.org/files/ENCFF560EBG/@@download/ENCFF560EBG.bed.gz
ENCFF046YUJ	ENCSR999NKW	https://www.encodeproject.org/files/ENCFF046YUJ/@@download/ENCFF046YUJ.bed.gz
ENCFF963ISM	ENCSR999NKW	https://www.encodeproject.org/files/ENCFF963ISM/@@download/ENCFF963ISM.bed.gz
ENCFF271ROE	ENCSR555ZDH	https://www.encodeproject.org/files/ENCFF271ROE/@@download/ENCFF271ROE.bed.gz
ENCFF232ELL	ENCSR177JWR	https://www.encodeproject.org/files/ENCFF232ELL/@@download/ENCFF232ELL.bed.gz
ENCFF984UZN	ENCSR032RGS	https://www.encodeproject.org/files/ENCFF984UZN/@@download/ENCFF984UZN.bed.gz
ENCFF127KOF	ENCSR032RGS	https://www.encodeproject.org/files/ENCFF127KOF/@@download/ENCFF127KOF.bed.gz

ENCFF548PSN	ENCSR032RGS	https://www.encodeproject.org/files/ENCFF548PSN/@@download/ENCFF548PSN.bed.gz
ENCFF063OBU	ENCSR032RGS	https://www.encodeproject.org/files/ENCFF063OBU/@@download/ENCFF063OBU.bed.gz
ENCFF479TER	ENCSR032RGS	https://www.encodeproject.org/files/ENCFF479TER/@@download/ENCFF479TER.bed.gz
ENCFF296VFY	ENCSR032RGS	https://www.encodeproject.org/files/ENCFF296VFY/@@download/ENCFF296VFY.bed.gz
ENCFF069IWR	ENCSR032RGS	https://www.encodeproject.org/files/ENCFF069IWR/@@download/ENCFF069IWR.bed.gz
ENCFF797FLV	ENCSR970UNF	https://www.encodeproject.org/files/ENCFF797FLV/@@download/ENCFF797FLV.bed.gz
ENCFF249EQM	ENCSR970UNF	https://www.encodeproject.org/files/ENCFF249EQM/@@download/ENCFF249EQM.bed.gz
ENCFF901IWE	ENCSR970UNF	https://www.encodeproject.org/files/ENCFF901IWE/@@download/ENCFF901IWE.bed.gz
ENCFF159YDR	ENCSR970UNF	https://www.encodeproject.org/files/ENCFF159YDR/@@download/ENCFF159YDR.bed.gz
ENCFF506RYJ	ENCSR310UDW	https://www.encodeproject.org/files/ENCFF506RYJ/@@download/ENCFF506RYJ.bed.gz
ENCFF845KMK	ENCSR157OSO	https://www.encodeproject.org/files/ENCFF845KMK/@@download/ENCFF845KMK.bed.gz
ENCFF257UAY	ENCSR157OSO	https://www.encodeproject.org/files/ENCFF257UAY/@@download/ENCFF257UAY.bed.gz
ENCFF285BXB	ENCSR017RQC	https://www.encodeproject.org/files/ENCFF994AHG/@@download/ENCFF994AHG.bed.gz
ENCFF374BNC	ENCSR017RQC	https://www.encodeproject.org/files/ENCFF956UCP/@@download/ENCFF956UCP.bed.gz
ENCFF305AHS	ENCSR823ZCR	https://www.encodeproject.org/files/ENCFF305AHS/@@download/ENCFF305AHS.bed.gz
ENCFF953QCT	ENCSR823ZCR	https://www.encodeproject.org/files/ENCFF953QCT/@@download/ENCFF953QCT.bed.gz
ENCFF998WFC	ENCSR337UIU	https://www.encodeproject.org/files/ENCFF998WFC/@@download/ENCFF998WFC.bed.gz
ENCFF244DRQ	ENCSR337UIU	https://www.encodeproject.org/files/ENCFF244DRQ/@@download/ENCFF244DRQ.bed.gz
ENCFF466CVI	ENCSR499ASS	https://www.encodeproject.org/files/ENCFF466CVI/@@download/ENCFF466CVI.bed.gz
ENCFF061YKV	ENCSR499ASS	https://www.encodeproject.org/files/ENCFF061YKV/@@download/ENCFF061YKV.bed.gz
ENCFF742PNL	ENCSR499ASS	https://www.encodeproject.org/files/ENCFF742PNL/@@download/ENCFF742PNL.bed.gz
ENCFF369VMF	ENCSR499ASS	https://www.encodeproject.org/files/ENCFF369VMF/@@download/ENCFF369VMF.bed.gz
ENCFF224QAO	ENCSR695FLC	https://www.encodeproject.org/files/ENCFF224QAO/@@download/ENCFF224QAO.bed.gz
ENCFF037ANG	ENCSR695FLC	https://www.encodeproject.org/files/ENCFF037ANG/@@download/ENCFF037ANG.bed.gz
ENCFF544MQW	ENCSR591PIX	https://www.encodeproject.org/files/ENCFF544MQW/@@download/ENCFF544MQW.bed.gz
ENCFF510OSH	ENCSR591PIX	https://www.encodeproject.org/files/ENCFF510OSH/@@download/ENCFF510OSH.bed.gz
ENCFF481ELM	ENCSR591PIX	https://www.encodeproject.org/files/ENCFF481ELM/@@download/ENCFF481ELM.bed.gz
ENCFF953NZY	ENCSR591PIX	https://www.encodeproject.org/files/ENCFF953NZY/@@download/ENCFF953NZY.bed.gz
ENCFF188MZK	ENCSR600ZHS	https://www.encodeproject.org/files/ENCFF188MZK/@@download/ENCFF188MZK.bed.gz
ENCFF993UXH	ENCSR600ZHS	https://www.encodeproject.org/files/ENCFF993UXH/@@download/ENCFF993UXH.bed.gz
ENCFF138ISY	ENCSR682ETE	https://www.encodeproject.org/files/ENCFF138ISY/@@download/ENCFF138ISY.bed.gz

ENCFF842EAT	ENCSR062SVK	https://www.encodeproject.org/files/ENCFF842EAT/@@download/ENCFF842EAT.bed.gz
ENCFF675UHQ	ENCSR062SVK	https://www.encodeproject.org/files/ENCFF675UHQ/@@download/ENCFF675UHQ.bed.gz
ENCFF524FYY	ENCSR490MSG	https://www.encodeproject.org/files/ENCFF524FYY/@@download/ENCFF524FYY.bed.gz
ENCFF646EYO	ENCSR490MSG	https://www.encodeproject.org/files/ENCFF646EYO/@@download/ENCFF646EYO.bed.gz
ENCFF563MKC	ENCSR548QCP	https://www.encodeproject.org/files/ENCFF563MKC/@@download/ENCFF563MKC.bed.gz
ENCFF207QOW	ENCSR548QCP	https://www.encodeproject.org/files/ENCFF207QOW/@@download/ENCFF207QOW.bed.gz
ENCFF929EDK	ENCSR204SMO	https://www.encodeproject.org/files/ENCFF929EDK/@@download/ENCFF929EDK.bed.gz
ENCFF792EIF	ENCSR204SMO	https://www.encodeproject.org/files/ENCFF792EIF/@@download/ENCFF792EIF.bed.gz
ENCFF421HWL	ENCSR542RNG	https://www.encodeproject.org/files/ENCFF421HWL/@@download/ENCFF421HWL.bed.gz
ENCFF481MCI	ENCSR542RNG	https://www.encodeproject.org/files/ENCFF481MCI/@@download/ENCFF481MCI.bed.gz
ENCFF613NGC	ENCSR303PWB	https://www.encodeproject.org/files/ENCFF613NGC/@@download/ENCFF613NGC.bed.gz
ENCFF254OVS	ENCSR303PWB	https://www.encodeproject.org/files/ENCFF254OVS/@@download/ENCFF254OVS.bed.gz
ENCFF620VTJ	ENCSR212LAZ	https://www.encodeproject.org/files/ENCFF620VTJ/@@download/ENCFF620VTJ.bed.gz
ENCFF423JET	ENCSR212LAZ	https://www.encodeproject.org/files/ENCFF423JET/@@download/ENCFF423JET.bed.gz
ENCFF072LFQ	ENCSR286STX	https://www.encodeproject.org/files/ENCFF072LFQ/@@download/ENCFF072LFQ.bed.gz
ENCFF122SGC	ENCSR584AXZ	https://www.encodeproject.org/files/ENCFF122SGC/@@download/ENCFF122SGC.bed.gz
ENCFF231FCU	ENCSR584AXZ	https://www.encodeproject.org/files/ENCFF231FCU/@@download/ENCFF231FCU.bed.gz
ENCFF560QLT	ENCSR390SLL	https://www.encodeproject.org/files/ENCFF560QLT/@@download/ENCFF560QLT.bed.gz
ENCFF024FSS	ENCSR390SLL	https://www.encodeproject.org/files/ENCFF024FSS/@@download/ENCFF024FSS.bed.gz
ENCFF778HMH	ENCSR948MQC	https://www.encodeproject.org/files/ENCFF778HMH/@@download/ENCFF778HMH.bed.gz
ENCFF718VNX	ENCSR485TLP	https://www.encodeproject.org/files/ENCFF718VNX/@@download/ENCFF718VNX.bed.gz
ENCFF930TUO	ENCSR485TLP	https://www.encodeproject.org/files/ENCFF930TUO/@@download/ENCFF930TUO.bed.gz
ENCFF330KWE	ENCSR485TLP	https://www.encodeproject.org/files/ENCFF330KWE/@@download/ENCFF330KWE.bed.gz
ENCFF067WME	ENCSR485TLP	https://www.encodeproject.org/files/ENCFF067WME/@@download/ENCFF067WME.bed.gz
ENCFF814ETV	ENCSR485TLP	https://www.encodeproject.org/files/ENCFF814ETV/@@download/ENCFF814ETV.bed.gz
ENCFF981DWW	ENCSR485TLP	https://www.encodeproject.org/files/ENCFF981DWW/@@download/ENCFF981DWW.bed.gz
ENCFF086BQP	ENCSR485TLP	https://www.encodeproject.org/files/ENCFF086BQP/@@download/ENCFF086BQP.bed.gz
ENCFF851GXJ	ENCSR765MXG	https://www.encodeproject.org/files/ENCFF851GXJ/@@download/ENCFF851GXJ.bed.gz
ENCFF883WHT	ENCSR765MXG	https://www.encodeproject.org/files/ENCFF883WHT/@@download/ENCFF883WHT.bed.gz
ENCFF950PSB	ENCSR914DTI	https://www.encodeproject.org/files/ENCFF950PSB/@@download/ENCFF950PSB.bed.gz
ENCFF871QBR	ENCSR637XSC	https://www.encodeproject.org/files/ENCFF871QBR/@@download/ENCFF871QBR.bed.gz

ENCFF223HZQ	ENCSR637XSC	https://www.encodeproject.org/files/ENCFF223HZQ/@@download/ENCFF223HZQ.bed.gz
ENCFF287FYP	ENCSR637XSC	https://www.encodeproject.org/files/ENCFF287FYP/@@download/ENCFF287FYP.bed.gz
ENCFF912ESR	ENCSR637XSC	https://www.encodeproject.org/files/ENCFF912ESR/@@download/ENCFF912ESR.bed.gz
ENCFF172DEA	ENCSR637XSC	https://www.encodeproject.org/files/ENCFF172DEA/@@download/ENCFF172DEA.bed.gz
ENCFF348YMZ	ENCSR637XSC	https://www.encodeproject.org/files/ENCFF348YMZ/@@download/ENCFF348YMZ.bed.gz
ENCFF713HRW	ENCSR637XSC	https://www.encodeproject.org/files/ENCFF713HRW/@@download/ENCFF713HRW.bed.gz
ENCFF342MRG	ENCSR291GJU	https://www.encodeproject.org/files/ENCFF342MRG/@@download/ENCFF342MRG.bed.gz
ENCFF455ILU	ENCSR291GJU	https://www.encodeproject.org/files/ENCFF455ILU/@@download/ENCFF455ILU.bed.gz
ENCFF828BPY	ENCSR291GJU	https://www.encodeproject.org/files/ENCFF828BPY/@@download/ENCFF828BPY.bed.gz
ENCFF356TXH	ENCSR291GJU	https://www.encodeproject.org/files/ENCFF356TXH/@@download/ENCFF356TXH.bed.gz
ENCFF415KLW	ENCSR291GJU	https://www.encodeproject.org/files/ENCFF415KLW/@@download/ENCFF415KLW.bed.gz
ENCFF295AJP	ENCSR291GJU	https://www.encodeproject.org/files/ENCFF295AJP/@@download/ENCFF295AJP.bed.gz
ENCFF759RMS	ENCSR291GJU	https://www.encodeproject.org/files/ENCFF759RMS/@@download/ENCFF759RMS.bed.gz
ENCFF068ZZZ	ENCSR647AOY	https://www.encodeproject.org/files/ENCFF068ZZZ/@@download/ENCFF068ZZZ.bed.gz
ENCFF172VSG	ENCSR846VLJ	https://www.encodeproject.org/files/ENCFF172VSG/@@download/ENCFF172VSG.bed.gz
ENCFF487NCC	ENCSR846VLJ	https://www.encodeproject.org/files/ENCFF487NCC/@@download/ENCFF487NCC.bed.gz
ENCFF887ARA	ENCSR654UYP	https://www.encodeproject.org/files/ENCFF887ARA/@@download/ENCFF887ARA.bed.gz
ENCFF970MTO	ENCSR654UYP	https://www.encodeproject.org/files/ENCFF970MTO/@@download/ENCFF970MTO.bed.gz
ENCFF816THB	ENCSR705KEB	https://www.encodeproject.org/files/ENCFF816THB/@@download/ENCFF816THB.bed.gz
ENCFF360KWB	ENCSR705KEB	https://www.encodeproject.org/files/ENCFF360KWB/@@download/ENCFF360KWB.bed.gz
ENCFF964DAT	ENCSR515CDW	https://www.encodeproject.org/files/ENCFF964DAT/@@download/ENCFF964DAT.bed.gz
ENCFF072PVO	ENCSR515CDW	https://www.encodeproject.org/files/ENCFF072PVO/@@download/ENCFF072PVO.bed.gz
ENCFF719IDZ	ENCSR540BML	https://www.encodeproject.org/files/ENCFF719IDZ/@@download/ENCFF719IDZ.bed.gz
ENCFF463WUA	ENCSR540BML	https://www.encodeproject.org/files/ENCFF463WUA/@@download/ENCFF463WUA.bed.gz
ENCFF925JFY	ENCSR689SDA	https://www.encodeproject.org/files/ENCFF925JFY/@@download/ENCFF925JFY.bed.gz
ENCFF666UUK	ENCSR689SDA	https://www.encodeproject.org/files/ENCFF666UUK/@@download/ENCFF666UUK.bed.gz
ENCFF951NYI	ENCSR096BPX	https://www.encodeproject.org/files/ENCFF951NYI/@@download/ENCFF951NYI.bed.gz
ENCFF984VPG	ENCSR096BPX	https://www.encodeproject.org/files/ENCFF984VPG/@@download/ENCFF984VPG.bed.gz
ENCFF849LPY	ENCSR774NXA	https://www.encodeproject.org/files/ENCFF849LPY/@@download/ENCFF849LPY.bed.gz
ENCFF203KWW	ENCSR774NXA	https://www.encodeproject.org/files/ENCFF203KWW/@@download/ENCFF203KWW.bed.gz
ENCFF590JMZ	ENCSR522FGI	https://www.encodeproject.org/files/ENCFF590JMZ/@@download/ENCFF590JMZ.bed.gz

ENCFF567VUC	ENCSR522FGI	https://www.encodeproject.org/files/ENCFF567VUC/@@download/ENCFF567VUC.bed.gz
ENCFF802ITJ	ENCSR710SMN	https://www.encodeproject.org/files/ENCFF802ITJ/@@download/ENCFF802ITJ.bed.gz
ENCFF556YGF	ENCSR710SMN	https://www.encodeproject.org/files/ENCFF556YGF/@@download/ENCFF556YGF.bed.gz
ENCFF633SCY	ENCSR955JSO	https://www.encodeproject.org/files/ENCFF633SCY/@@download/ENCFF633SCY.bed.gz
ENCFF738IZU	ENCSR955JSO	https://www.encodeproject.org/files/ENCFF738IZU/@@download/ENCFF738IZU.bed.gz
ENCFF381CCN	ENCSR918TVE	https://www.encodeproject.org/files/ENCFF381CCN/@@download/ENCFF381CCN.bed.gz
ENCFF176EOV	ENCSR918TVE	https://www.encodeproject.org/files/ENCFF176EOV/@@download/ENCFF176EOV.bed.gz
ENCFF870OMG	ENCSR630REB	https://www.encodeproject.org/files/ENCFF870OMG/@@download/ENCFF870OMG.bed.gz
ENCFF831JKU	ENCSR630REB	https://www.encodeproject.org/files/ENCFF831JKU/@@download/ENCFF831JKU.bed.gz
ENCFF126OSM	ENCSR864ADD	https://www.encodeproject.org/files/ENCFF126OSM/@@download/ENCFF126OSM.bed.gz
ENCFF967XFP	ENCSR864ADD	https://www.encodeproject.org/files/ENCFF967XFP/@@download/ENCFF967XFP.bed.gz
ENCFF917REN	ENCSR095QNB	https://www.encodeproject.org/files/ENCFF917REN/@@download/ENCFF917REN.bed.gz
ENCFF945SYZ	ENCSR095QNB	https://www.encodeproject.org/files/ENCFF945SYZ/@@download/ENCFF945SYZ.bed.gz
ENCFF614SMH	ENCSR095QNB	https://www.encodeproject.org/files/ENCFF614SMH/@@download/ENCFF614SMH.bed.gz
ENCFF346CZA	ENCSR095QNB	https://www.encodeproject.org/files/ENCFF346CZA/@@download/ENCFF346CZA.bed.gz
ENCFF316BML	ENCSR773USD	https://www.encodeproject.org/files/ENCFF316BML/@@download/ENCFF316BML.bed.gz
ENCFF075WWH	ENCSR773USD	https://www.encodeproject.org/files/ENCFF075WWH/@@download/ENCFF075WWH.bed.gz
ENCFF319FIY	ENCSR386HAZ	https://www.encodeproject.org/files/ENCFF319FIY/@@download/ENCFF319FIY.bed.gz
ENCFF609YMS	ENCSR386HAZ	https://www.encodeproject.org/files/ENCFF609YMS/@@download/ENCFF609YMS.bed.gz
ENCFF408QRF	ENCSR491VXJ	https://www.encodeproject.org/files/ENCFF408QRF/@@download/ENCFF408QRF.bed.gz
ENCFF464HPU	ENCSR491VXJ	https://www.encodeproject.org/files/ENCFF464HPU/@@download/ENCFF464HPU.bed.gz
ENCFF525ZRG	ENCSR310RJN	https://www.encodeproject.org/files/ENCFF525ZRG/@@download/ENCFF525ZRG.bed.gz
ENCFF308DNB	ENCSR846ZBX	https://www.encodeproject.org/files/ENCFF308DNB/@@download/ENCFF308DNB.bed.gz
ENCFF544RPU	ENCSR846ZBX	https://www.encodeproject.org/files/ENCFF544RPU/@@download/ENCFF544RPU.bed.gz
ENCFF780NPL	ENCSR260ZIV	https://www.encodeproject.org/files/ENCFF780NPL/@@download/ENCFF780NPL.bed.gz
ENCFF580UXE	ENCSR260ZIV	https://www.encodeproject.org/files/ENCFF580UXE/@@download/ENCFF580UXE.bed.gz
ENCFF898WYV	ENCSR133CMC	https://www.encodeproject.org/files/ENCFF898WYV/@@download/ENCFF898WYV.bed.gz
ENCFF889SBW	ENCSR651SOJ	https://www.encodeproject.org/files/ENCFF889SBW/@@download/ENCFF889SBW.bed.gz
ENCFF389YDC	ENCSR651SOJ	https://www.encodeproject.org/files/ENCFF389YDC/@@download/ENCFF389YDC.bed.gz
ENCFF520ASW	ENCSR074WMH	https://www.encodeproject.org/files/ENCFF520ASW/@@download/ENCFF520ASW.bed.gz
ENCFF184BRH	ENCSR074WMH	https://www.encodeproject.org/files/ENCFF184BRH/@@download/ENCFF184BRH.bed.gz

ENCFF873MVF	ENCSR437OOJ	https://www.encodeproject.org/files/ENCFF873MVF/@@download/ENCFF873MVF.bed.gz
ENCFF315UYG	ENCSR437OOJ	https://www.encodeproject.org/files/ENCFF315UYG/@@download/ENCFF315UYG.bed.gz
ENCFF179JFB	ENCSR053SGP	https://www.encodeproject.org/files/ENCFF179JFB/@@download/ENCFF179JFB.bed.gz
ENCFF605QTK	ENCSR808ZMK	https://www.encodeproject.org/files/ENCFF605QTK/@@download/ENCFF605QTK.bed.gz
ENCFF201EYO	ENCSR808ZMK	https://www.encodeproject.org/files/ENCFF201EYO/@@download/ENCFF201EYO.bed.gz
ENCFF112NVU	ENCSR117PYB	https://www.encodeproject.org/files/ENCFF112NVU/@@download/ENCFF112NVU.bed.gz
ENCFF174HPZ	ENCSR117PYB	https://www.encodeproject.org/files/ENCFF174HPZ/@@download/ENCFF174HPZ.bed.gz
ENCFF144VSH	ENCSR213YPO	https://www.encodeproject.org/files/ENCFF144VSH/@@download/ENCFF144VSH.bed.gz
ENCFF783MYY	ENCSR213YPO	https://www.encodeproject.org/files/ENCFF783MYY/@@download/ENCFF783MYY.bed.gz
ENCFF298TYK	ENCSR258JCL	https://www.encodeproject.org/files/ENCFF298TYK/@@download/ENCFF298TYK.bed.gz
ENCFF034WCY	ENCSR258JCL	https://www.encodeproject.org/files/ENCFF034WCY/@@download/ENCFF034WCY.bed.gz
ENCFF773CEZ	ENCSR373TDL	https://www.encodeproject.org/files/ENCFF773CEZ/@@download/ENCFF773CEZ.bed.gz
ENCFF522FNJ	ENCSR373TDL	https://www.encodeproject.org/files/ENCFF522FNJ/@@download/ENCFF522FNJ.bed.gz
ENCFF199VHV	ENCSR563ZNI	https://www.encodeproject.org/files/ENCFF199VHV/@@download/ENCFF199VHV.bed.gz
ENCFF455PLR	ENCSR788TRR	https://www.encodeproject.org/files/ENCFF455PLR/@@download/ENCFF455PLR.bed.gz
ENCFF963YOX	ENCSR788TRR	https://www.encodeproject.org/files/ENCFF963YOX/@@download/ENCFF963YOX.bed.gz
ENCFF431CPR	ENCSR072UYN	https://www.encodeproject.org/files/ENCFF431CPR/@@download/ENCFF431CPR.bed.gz
ENCFF277OGS	ENCSR072UYN	https://www.encodeproject.org/files/ENCFF277OGS/@@download/ENCFF277OGS.bed.gz
ENCFF203EQG	ENCSR152PSA	https://www.encodeproject.org/files/ENCFF203EQG/@@download/ENCFF203EQG.bed.gz
ENCFF804EIN	ENCSR152PSA	https://www.encodeproject.org/files/ENCFF804EIN/@@download/ENCFF804EIN.bed.gz
ENCFF873GXL	ENCSR210NKB	https://www.encodeproject.org/files/ENCFF873GXL/@@download/ENCFF873GXL.bed.gz
ENCFF643ZJJ	ENCSR210NKB	https://www.encodeproject.org/files/ENCFF643ZJJ/@@download/ENCFF643ZJJ.bed.gz
ENCFF305GIG	ENCSR868FGK	https://www.encodeproject.org/files/ENCFF305GIG/@@download/ENCFF305GIG.bed.gz
ENCFF760HHY	ENCSR868FGK	https://www.encodeproject.org/files/ENCFF760HHY/@@download/ENCFF760HHY.bed.gz
ENCFF545AQB	ENCSR868FGK	https://www.encodeproject.org/files/ENCFF545AQB/@@download/ENCFF545AQB.bed.gz
ENCFF222VTK	ENCSR868FGK	https://www.encodeproject.org/files/ENCFF222VTK/@@download/ENCFF222VTK.bed.gz
ENCFF530FMG	ENCSR868FGK	https://www.encodeproject.org/files/ENCFF530FMG/@@download/ENCFF530FMG.bed.gz
ENCFF748OSQ	ENCSR868FGK	https://www.encodeproject.org/files/ENCFF748OSQ/@@download/ENCFF748OSQ.bed.gz
ENCFF966AKO	ENCSR868FGK	https://www.encodeproject.org/files/ENCFF966AKO/@@download/ENCFF966AKO.bed.gz
ENCFF912MCC	ENCSR516CPW	https://www.encodeproject.org/files/ENCFF912MCC/@@download/ENCFF912MCC.bed.gz
ENCFF300UPI	ENCSR846VPV	https://www.encodeproject.org/files/ENCFF300UPI/@@download/ENCFF300UPI.bed.gz

ENCFF303AKJ	ENCSR846VPV	https://www.encodeproject.org/files/ENCFF303AKJ/@@download/ENCFF303AKJ.bed.gz
ENCFF882FLC	ENCSR422SUG	https://www.encodeproject.org/files/ENCFF882FLC/@@download/ENCFF882FLC.bed.gz
ENCFF204NII	ENCSR422SUG	https://www.encodeproject.org/files/ENCFF204NII/@@download/ENCFF204NII.bed.gz
ENCFF882OVP	ENCSR422SUG	https://www.encodeproject.org/files/ENCFF882OVP/@@download/ENCFF882OVP.bed.gz
ENCFF905GGG	ENCSR422SUG	https://www.encodeproject.org/files/ENCFF905GGG/@@download/ENCFF905GGG.bed.gz
ENCFF885TNX	ENCSR308HPZ	https://www.encodeproject.org/files/ENCFF885TNX/@@download/ENCFF885TNX.bed.gz
ENCFF461RJQ	ENCSR308HPZ	https://www.encodeproject.org/files/ENCFF461RJQ/@@download/ENCFF461RJQ.bed.gz
ENCFF791RKW	ENCSR042AWH	https://www.encodeproject.org/files/ENCFF791RKW/@@download/ENCFF791RKW.bed.gz
ENCFF851SSE	ENCSR042AWH	https://www.encodeproject.org/files/ENCFF851SSE/@@download/ENCFF851SSE.bed.gz
ENCFF314RIW	ENCSR042AWH	https://www.encodeproject.org/files/ENCFF314RIW/@@download/ENCFF314RIW.bed.gz
ENCFF438JMM	ENCSR042AWH	https://www.encodeproject.org/files/ENCFF438JMM/@@download/ENCFF438JMM.bed.gz
ENCFF133HUE	ENCSR040DJK	https://www.encodeproject.org/files/ENCFF133HUE/@@download/ENCFF133HUE.bed.gz
ENCFF784MGH	ENCSR040DJK	https://www.encodeproject.org/files/ENCFF784MGH/@@download/ENCFF784MGH.bed.gz
ENCFF278WQH	ENCSR201FIW	https://www.encodeproject.org/files/ENCFF278WQH/@@download/ENCFF278WQH.bed.gz
ENCFF675XYC	ENCSR201FIW	https://www.encodeproject.org/files/ENCFF675XYC/@@download/ENCFF675XYC.bed.gz
ENCFF356UPB	ENCSR551CSY	https://www.encodeproject.org/files/ENCFF356UPB/@@download/ENCFF356UPB.bed.gz
ENCFF774JIK	ENCSR551CSY	https://www.encodeproject.org/files/ENCFF774JIK/@@download/ENCFF774JIK.bed.gz
ENCFF193HEJ	ENCSR551CSY	https://www.encodeproject.org/files/ENCFF193HEJ/@@download/ENCFF193HEJ.bed.gz
ENCFF164FNM	ENCSR551CSY	https://www.encodeproject.org/files/ENCFF164FNM/@@download/ENCFF164FNM.bed.gz
ENCFF049BCQ	ENCSR551CSY	https://www.encodeproject.org/files/ENCFF049BCQ/@@download/ENCFF049BCQ.bed.gz
ENCFF385QXA	ENCSR551CSY	https://www.encodeproject.org/files/ENCFF385QXA/@@download/ENCFF385QXA.bed.gz
ENCFF368MPK	ENCSR551CSY	https://www.encodeproject.org/files/ENCFF368MPK/@@download/ENCFF368MPK.bed.gz
ENCFF169RTS	ENCSR551CSY	https://www.encodeproject.org/files/ENCFF169RTS/@@download/ENCFF169RTS.bed.gz
ENCFF912IDR	ENCSR607BTF	https://www.encodeproject.org/files/ENCFF912IDR/@@download/ENCFF912IDR.bed.gz
ENCFF347HUL	ENCSR607BTF	https://www.encodeproject.org/files/ENCFF347HUL/@@download/ENCFF347HUL.bed.gz
ENCFF461IGU	ENCSR241OBO	https://www.encodeproject.org/files/ENCFF461IGU/@@download/ENCFF461IGU.bed.gz
ENCFF315XZD	ENCSR241OBO	https://www.encodeproject.org/files/ENCFF315XZD/@@download/ENCFF315XZD.bed.gz
ENCFF679YNL	ENCSR404LLJ	https://www.encodeproject.org/files/ENCFF679YNL/@@download/ENCFF679YNL.bed.gz
ENCFF995XLK	ENCSR404LLJ	https://www.encodeproject.org/files/ENCFF995XLK/@@download/ENCFF995XLK.bed.gz
ENCFF913BUA	ENCSR831KAH	https://www.encodeproject.org/files/ENCFF913BUA/@@download/ENCFF913BUA.bed.gz
ENCFF623GOH	ENCSR831KAH	https://www.encodeproject.org/files/ENCFF623GOH/@@download/ENCFF623GOH.bed.gz

ENCFF510PGR	ENCSR761TKU	https://www.encodeproject.org/files/ENCFF510PGR/@@download/ENCFF510PGR.bed.gz
ENCFF668ARP	ENCSR761TKU	https://www.encodeproject.org/files/ENCFF668ARP/@@download/ENCFF668ARP.bed.gz
ENCFF235JZB	ENCSR200OML	https://www.encodeproject.org/files/ENCFF235JZB/@@download/ENCFF235JZB.bed.gz
ENCFF383XOM	ENCSR200OML	https://www.encodeproject.org/files/ENCFF383XOM/@@download/ENCFF383XOM.bed.gz
ENCFF114GDS	ENCSR200OML	https://www.encodeproject.org/files/ENCFF114GDS/@@download/ENCFF114GDS.bed.gz
ENCFF982UNH	ENCSR200OML	https://www.encodeproject.org/files/ENCFF982UNH/@@download/ENCFF982UNH.bed.gz
ENCFF329NTV	ENCSR996ZCR	https://www.encodeproject.org/files/ENCFF329NTV/@@download/ENCFF329NTV.bed.gz
ENCFF250ANB	ENCSR996ZCR	https://www.encodeproject.org/files/ENCFF250ANB/@@download/ENCFF250ANB.bed.gz
ENCFF775HDQ	ENCSR153IUB	https://www.encodeproject.org/files/ENCFF775HDQ/@@download/ENCFF775HDQ.bed.gz
ENCFF173AVZ	ENCSR153IUB	https://www.encodeproject.org/files/ENCFF173AVZ/@@download/ENCFF173AVZ.bed.gz
ENCFF816GCG	ENCSR670REK	https://www.encodeproject.org/files/ENCFF816GCG/@@download/ENCFF816GCG.bed.gz
ENCFF456JOB	ENCSR670REK	https://www.encodeproject.org/files/ENCFF456JOB/@@download/ENCFF456JOB.bed.gz
ENCFF822SDP	ENCSR851SBY	https://www.encodeproject.org/files/ENCFF822SDP/@@download/ENCFF822SDP.bed.gz
ENCFF430VEQ	ENCSR851SBY	https://www.encodeproject.org/files/ENCFF430VEQ/@@download/ENCFF430VEQ.bed.gz

Table S2 Computing Power and Processing Time for Each Model

**Computation power**	**Model**	**PBMC**	**Brain**
One GPU	**scEpiLock**	1:39:53	4:14:56
	DeepSEA	1:06:06	2:47:59
	DanQ	1:19:02	3:23:34
One CPU	Random forest	0:06:25	0:07:54

Table S3 H3K27ac Enrichment in Key and Non-key Regions

Data	Region	H3K27ac enrichment	p-value
PBMC	key	0.554	<2.2e-16
	Non-key	0.412	
Brain	key	1.448	<2.2e-16
	Non-key	0.987	
